# Prognostic value of the neutrophil percentage-to-albumin ratio for mortality in patients with hyperuricemia and gout

**DOI:** 10.1186/s12872-026-06251-4

**Published:** 2026-07-17

**Authors:** Xia-Ying Xu, Da-Hai Li, Qing Ou, Yu-Cheng Li, Hong-Yun Wei, Tong Sha

**Affiliations:** 1 Department of Critical Care Medicine, Nanfang Hospital, Southern Medical University, Guangzhou, 510515 China; 2 Department of Dialysis Center, Nanfang Hospital, Southern Medical University, Guangzhou, 510515 China

**Keywords:** Neutrophil percentage-to-albumin ratio, Hyperuricemia, Gout, All-cause mortality, Cardiovascular mortality

## Abstract

**Background:**

The neutrophil percentage-to-albumin ratio (NPAR) is a composite biomarker reflecting systemic inflammation and nutritional imbalance. Its prognostic significance for mortality in patients with hyperuricemia (HUA) or gout remains incompletely understood, and whether this association differs between asymptomatic HUA and gout has not been examined.

**Methods:**

We utilized data from the National Health and Nutrition Examination Survey (NHANES, 1999–2018). After applying exclusion criteria, 4,344 adults were identified, comprising 3,659 with asymptomatic hyperuricemia and 685 with gout (56.8% male; median age 52 years). The neutrophil percentage-to-albumin ratio (NPAR) was calculated for each participant. All analyses were stratified by clinical subgroup (asymptomatic hyperuricemia vs. gout). Associations between NPAR and all-cause and cardiovascular mortality were evaluated using Kaplan–Meier analysis, weighted multivariable Cox proportional hazards regression, and restricted cubic spline (RCS) models. To account for competing risks from non-cardiovascular death, Fine–Gray subdistribution hazard models were fitted, and cumulative incidence functions were estimated by the Aalen–Johansen method.

**Results:**

In the asymptomatic HUA subgroup, participants in the highest NPAR quartile (Q4) exhibited significantly elevated risks of all-cause mortality (adjusted HR = 2.08, 95% CI: 1.56–2.78, *P* < 0.001) and cardiovascular mortality (adjusted HR = 2.24, 95% CI: 1.41–3.56, *P* = 0.001) after full adjustment, compared with the lowest quartile (Q1). In the gout subgroup, the corresponding HR was 2.14 (*P* = 0.028) for all-cause mortality; for cardiovascular mortality, the quartile-based HR was 3.51 (*P* = 0.060), while RCS analysis showed a peak HR of 4.95 at the highest NPAR values. NPAR demonstrated strong predictive accuracy in the full cohort, with an AUC of 0.875 for both all-cause and cardiovascular mortality (Model 4, 10-year). In the competing risks analysis, the association between NPAR and cardiovascular mortality remained significant after accounting for non-cardiovascular death as a competing event, with Q4 maintaining an elevated risk across all adjustment levels (fully adjusted sHR = 1.899, 95% CI: 1.366–2.640, *P* < 0.001; P for trend < 0.001).

**Conclusion:**

NPAR is a robust independent predictor of all-cause and cardiovascular mortality in patients with asymptomatic HUA. The association was attenuated in the gout subgroup, likely due to the smaller sample size and limited cardiovascular events, likely reflecting the smaller sample size and limited cardiovascular events. NPAR may serve as a practical clinical biomarker for risk stratification in HUA and gout populations.

**Supplementary Information:**

The online version contains supplementary material available at https://doi.org/10.1186/s12872-026-06251-4.

## Introduction

Hyperuricemia (HUA) and gout are metabolic disorders resulting from disturbances in purine metabolism and represent a continuous pathological process [1]. These conditions are pervasive across geographic regions and are associated with substantially elevated risks of all-cause and cardiovascular mortality. Notably, HUA constitutes the fundamental pathogenic mechanism underlying gout, with its epidemiological burden demonstrating a conspicuous upward trend worldwide [2, 3]. Although uric acid-lowering therapies can alleviate symptoms, evidence from large-scale randomized trials and cohort studies [4–6] indicates that patients undergoing such treatments continue to face significantly heightened risks of cardiovascular complications and all-cause mortality. For instance, the Cardiovascular Safety of Febuxostat and Allopurinol in Patients with Gout (CARES) trial, a multicenter, double-blind, noninferiority trial involving 6,190 patients with gout and established cardiovascular disease, demonstrated that febuxostat was associated with increased cardiovascular mortality (HR 1.34, 95% CI 1.03–1.73) and all-cause mortality (HR 1.22, 95% CI 1.01–1.47) compared with allopurinol over a median follow-up of 32 months [4]. These findings were subsequently contextualized by the ALL-HEART trial, which compared allopurinol with usual care in patients with ischaemic heart disease and highlighted the potential cardiovascular benefits of urate-lowering therapy [7]. Furthermore, real-world cohort studies have consistently shown that despite urate-lowering therapy, patients with gout remain at elevated risk for cardiovascular events and mortality [5, 6]. The threat is notably higher in those suffering from chronic kidney disease or heart failure, among whom many patients still encounter elevated cardiovascular complications and all-cause mortality risks [4, 5]. A single measurement of serum uric acid is insufficient to accurately predict all-cause and cardiovascular mortality. While individual markers such as serum uric acid and CRP provide limited prognostic information, composite biomarkers integrating both inflammatory and nutritional dimensions remain underutilized in clinical practice. Therefore, more sensitive and comprehensive indicators are urgently needed to improve risk assessment [8, 9]. It is important to distinguish between asymptomatic HUA and gout, as they represent different stages of the same pathological continuum. In patients with gout, substantial evidence supports that urate-lowering therapy (ULT) reduces cardiovascular risk; for example, colchicine and febuxostat have demonstrated cardioprotective effects in gout populations [4, 10]. In contrast, among individuals with asymptomatic HUA, no randomized controlled trial has yet demonstrated that pharmacological urate reduction prevents subsequent cardiovascular events [11, 12]. This distinction underscores the need for separate prognostic assessment in these two populations.

Uric acid crystals are known to induce chronic inflammation, activate neutrophils to generate reactive oxygen species (ROS), and contribute to endothelial dysfunction and atherosclerosis [13]. As a dual marker of systemic inflammation, the neutrophil-to-albumin ratio (NPAR) is characterized by increased neutrophil levels and diminished albumin amounts [14]. A growing body of evidence has established the prognostic value of NPAR for mortality risk across diverse clinical populations. Liu et al. first reported that NPAR was associated with all-cause and cardiovascular mortality in the general US adult population, providing foundational evidence for its broad applicability as a prognostic biomarker [15]. Building upon these general population findings, subsequent studies have extended this evidence to specific disease contexts. In hypertensive patients, Yang et al. demonstrated that elevated NPAR predicted increased all-cause and cardiovascular mortality [16], while Li and Yu further confirmed its association with cerebrovascular mortality in this population [17]. In patients with diabetes, multiple studies have shown that higher NPAR levels are significantly associated with increased risks of all-cause and cardiovascular mortality [18–21]. Similarly, NPAR has been identified as an independent predictor of long-term mortality in patients with heart failure [22] and chronic kidney disease [23], as well as in cancer survivors [24]. These collective findings underscore NPAR’s broad applicability as a prognostic biomarker across various disease contexts.

Despite the established prognostic value of NPAR across diverse clinical populations, its utility in patients with hyperuricemia or gout—conditions characterized by chronic low-grade inflammation and elevated cardiovascular risk—remains unexplored. This gap is particularly notable given that both uric acid and NPAR contribute to vascular damage through inflammatory pathways, suggesting potential synergistic effects on mortality risk. Ji et al. demonstrated that serum uric acid partially mediated the association between NPAR and metabolic syndrome risk in a large US population-based study, with an indirect effect accounting for 14.93% of the total association, indicating a pathophysiological link between NPAR and uric acid metabolism. However, they did not evaluate the predictive capacity of NPAR for mortality in HUA or gout populations [25]. Unlike conventional markers such as serum uric acid and C-reactive protein (CRP), NPAR integrates both inflammatory and nutritional dimensions, offering a more comprehensive assessment of pathophysiological status [14, 26]. An increased NPAR signifies persistent neutrophil activation, which promotes the release of interleukin-6 (IL-6) within the vascular endothelium, thereby contributing to the destabilization of arterial plaques [14, 26]. Considering that both uric acid and NPAR contribute to vascular damage through inflammatory pathways, there exists the potential for a synergistic interaction that could substantially increase mortality risk. Kim et al. conducted a historical meta-analysis revealing that HUA increased the risk of stroke by 47% [27]; however, their study did not incorporate novel composite markers such as NPAR. In contrast, Han et al. identified an association between an NPAR value exceeding 13.28 and a hazard ratio (HR) of 1.74 for cardiovascular mortality in the general US adult population [9], yet their study was limited to participants from the general population and did not include a subgroup analysis specifically targeting HUA.

Therefore, the present study was designed to address this critical knowledge gap. This study aimed to investigate the correlation between NPAR and mortality outcomes (encompassing both general mortality and cardiovascular-related deaths) in patients with HUA and gout, based on a comprehensive dataset procured from the NHANES database during the 1999–2018 observational period. Furthermore, the research attempts to quantify the dose-response relationship between NPAR and mortality. To our knowledge, this is the first large-scale, population-based study to evaluate NPAR as a prognostic marker for mortality in individuals with HUA or gout, using nationally representative data from the United States. These findings may improve risk stratification of HUA patients and provide a robust evidence base for early interventions addressing inflammation and nutritional imbalances.

## Methods

### Study design and data source

The NHANES database, managed by the National Center for Health Statistics (NCHS), provided participants from 1999 to 2018. This dataset, refreshed biennially, comprises questionnaire data and clinical or laboratory assessments. For additional details, check out www.cdc.gov/nchs/nhanes/index.htm. The NHANES protocol was approved by the NCHS Research Ethics Review Board, and all participants provided written informed consent. As this study used publicly available de-identified data, additional ethical approval was not required.

### Participants selection

**Fig. 1:**
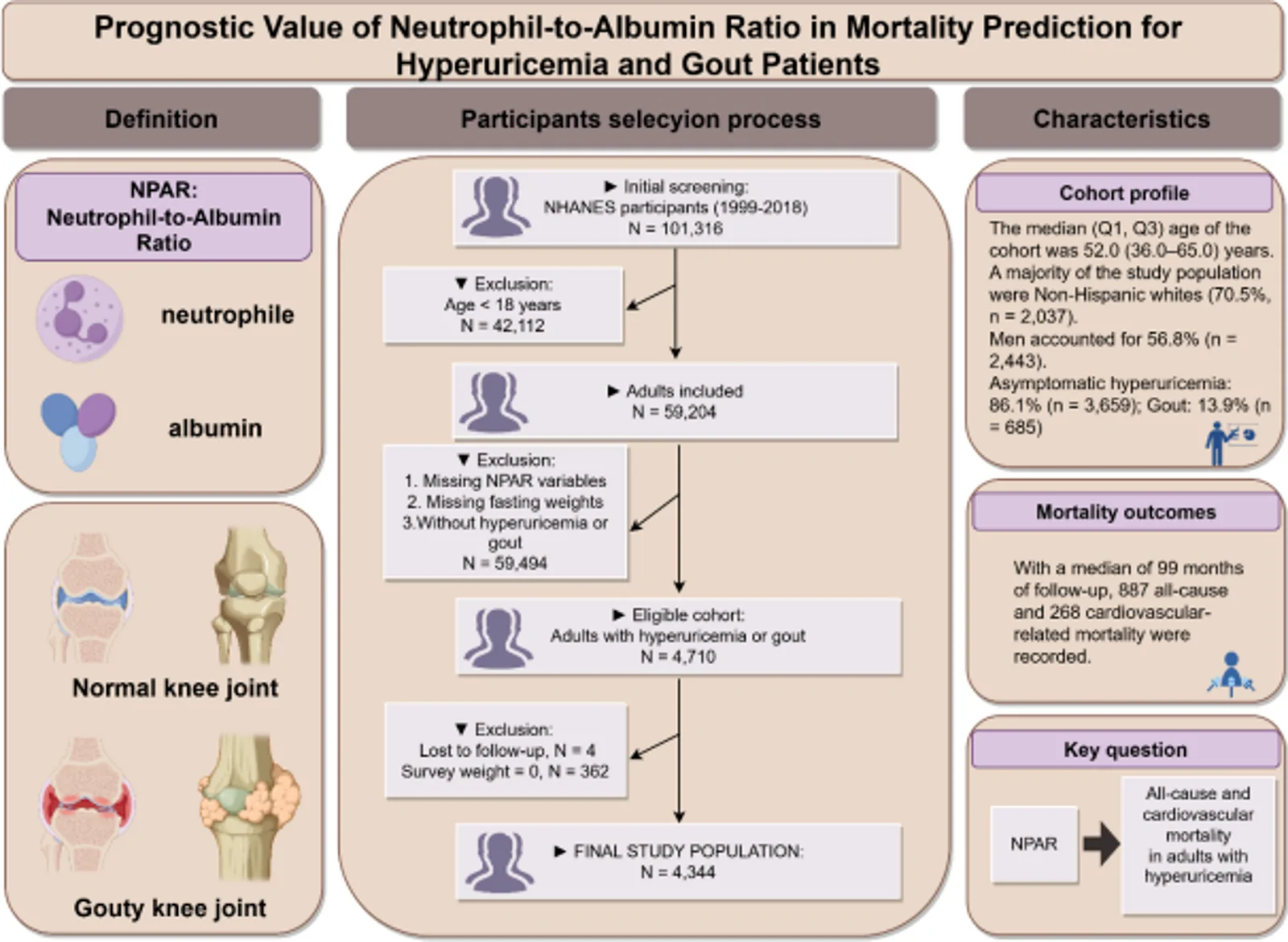
NPAR = neutrophil percentage-to-albumin ratio; NHANES = National Health and Nutrition Examination Survey. Adults with hyperuricemia were defined as serum uric acid ≥ 7.0 mg/dL (men) or ≥ 6.0 mg/dL (women), or self-reported gout. Participants with zero survey weights were excluded to ensure valid complex sampling variance estimation

The analysis sample group comprised 101,316 participants from ten biennial NHANES surveys conducted over the period from 1999 to 2018. Exclusion was based on the following criteria: (a) people younger than 18 years old, (b) participants with missing mortality data, (c) individuals without HUA or gout, where HUA is defined by serum uric acid levels of ≥ 420 µmol/L (7.0 mg/dL) for men and ≥ 360 µmol/L (6.0 mg/dL) for women, in accordance with established diagnostic standards, and (d) participants with incomplete hematological data (neutrophil count), biochemical data (albumin levels), or missing anthropometric measurements. The diagnosis of gout was affirmative responses to the question: “Has a doctor ever diagnosed you with gout?” The final sample for analysis, upon implementing these exclusion criteria, was made up of 4,344 eligible adults with complete data on exposure, outcome, and covariates. (see Fig. 1). In addition to the traditional diagnostic criteria for HUA, we conducted a sensitivity analysis using the cardiovascular-specific cutoff proposed by the URRAH study (≥ 5.6 mg/dL or ≥ 333 µmol/L for both sexes) [28, 29], which has been identified as the optimal threshold for predicting cardiovascular mortality in large cohort studies. The study population was further classified into asymptomatic HUA (*n* = 3,659) and gout (*n* = 685) subgroups.

### Measurement of NPAR

The percentage of neutrophils was quantified employing a Beckman Coulter analyzer, while **serum albumin concentration was measured using the bromocresol green (BCG) method, as specified in the NHANES laboratory procedures. NPAR was calculated as the ratio of neutrophil percentage (%) to albumin concentration (g/dL). Detailed descriptions of the laboratory procedures and quality control measures are available on the official NHANES website. During data analysis, participants with incomplete NPAR data were excluded. Statistical analyses used survey weights to address NHANES’s complex sampling design, ensuring nationally representative results.

### Mortality outcomes

The follow-up period is defined as the interval between the initial interview date and the death date, ascertained via the National Death Index (NDI) database ( https://www.cdc.gov/nchs/data-linkage/mortality-public ). We assessed the mortality rate of the study group by analyzing the survival outcomes of the participants, with the follow-up extending through December 31, 2019. Underlying causes of death were classified according to the International Classification of Diseases, 10th Revision (ICD-10).

### Covariate definitions

Demographic characteristics of participants with HUA and gout were extracted from the NHANES database. Socio-economic data were collected, encompassing aspects including race/ethnicity, gender, and age (categorized as Mexican American, Other Hispanic, non-Hispanic White, non-Hispanic Black, or other), educational attainment (defined as high school or less, some college, or more than a college degree), marital status (categorized as married/living with partner, widowed/divorced/separated, or never married), and the poverty income ratio (PIR, sorted into categories of below 1.3, between 1.3 and 3.5, or greater than 3.5). Additionally, comprehensive data on lifestyle factors and comorbidity histories were collected. Three categories were used to classify smoking status: never smokers (individuals who have smoked less than 100 cigarettes throughout their life), former smokers (people who have consumed more than 100 cigarettes and have now quit), and current smokers (those who have ever smoked over 100 cigarettes or are smoking every day now). Consumption of alcoholic drinks was classified into two categories: abstinence (no alcohol consumption in the past year) and alcohol consumption (frequency of intake over the preceding 12 months). Hypertension was defined as physician-diagnosed or measured systolic blood pressure ≥ 140 mmHg or diastolic blood pressure ≥ 90 mmHg. Diabetes was identified by the presence of one or more of the following criteria: (1) fasting blood glucose level ≥ 7 mmol/L, (2) a glycated hemoglobin (HbA1c) level of 6.5% or higher, (3) diabetes medication or insulin usage as reported by the individual, or (4) the identification of diabetes by a doctor. The presence of cancer and cardiovascular disease (CVD) was recorded as binary variables (yes or no). Rheumatic disease was defined as physician-diagnosed rheumatoid arthritis or psoriatic arthritis (MCQ195 = 2 or 3), with gout patients excluded to avoid overlap with the study population. Additional data were obtained through both physical and laboratory assessments, encompassing body mass index (BMI), uric acid levels (µmol/L), and the duration of follow-up. BMI was calculated as the ratio of an individual’s weight (in kilograms) to the square of their height (in meters), with classifications as follows: overweight (25.0–29.9 kg/m²) and obese (≥ 30.0 kg/m²). Cardiovascular medications were identified from the NHANES RXQ prescription module via detailed drug name matching, including ACEI/ARBs, β-blockers, calcium channel blockers (CCBs), diuretics, statins, antiplatelet agents, antidiabetic agents, and urate-lowering therapy (ULT). For both all-cause and cardiovascular mortality endpoints, we employed an identical set of covariates in the multivariable models.

To determine the covariates to be included in the multivariable model, we constructed a directed acyclic graph (DAG) using the dagitty R package (version 4.2.3) to identify the minimal sufficient adjustment set. The DAG was built based on existing literature and clinical knowledge, specifying the causal directions among the exposure (HUA/gout), outcomes (all-cause and cardiovascular mortality), and potential confounders. Through DAG analysis, we identified the set of confounders requiring adjustment, including demographic characteristics (age, sex, race/ethnicity), socioeconomic factors (education, marital status, PIR), lifestyle factors (smoking, alcohol consumption), comorbidities (hypertension, diabetes, CVD, cancer, rheumatic disease), and laboratory and physical examination indicators (BMI, total cholesterol, LDL cholesterol, HbA1c, fasting glucose, eGFR, systolic blood pressure, diastolic blood pressure). Additionally, to account for survey design and follow-up duration, survey year and follow-up time (i.e., number of participants at risk, for time-dependent analyses) were included in the model (see Fig. S8).

To assess multicollinearity among covariates in the multivariable model, we calculated the generalized variance inflation factor (GVIF) and its degrees-of-freedom-adjusted form, GVIF^(1/(2×Df)). This adjusted form is appropriate for categorical variables and effectively corrects for differences in degrees of freedom. The severity of multicollinearity was classified as follows: severe (GVIF^(1/(2×Df)) ≥ 2.0), moderate (1.5–2.0), mild (1.2–1.5), and none (< 1.2). The results showed that all variables had GVIF^(1/(2×Df)) values below 2.0, indicating no severe multicollinearity. As shown in Table S1, no variable exceeded the critical threshold of 2.0, indicating acceptable multicollinearity levels. Given the adequate sample size and that no variable exceeded the critical threshold of 2.0, all variables were retained in the final model (see Table S1).

### Statistical analysis

All analyses incorporated NHANES sample weights and stratification parameters to account for the complex multistage sampling design and ensure nationally representative estimates. Participants were divided into four NPAR quartiles: Q1 (NPAR ≤ 12.20), Q2 (12.20 < NPAR ≤ 13.89), Q3 (13.90 < NPAR ≤ 15.69), and Q4 (NPAR ≥ 15.70). To determine the normality of each variable’s distribution, the Kolmogorov‑Smirnov test was applied. As shown in Fig. S1, none of the continuous variables displayed normal distributions. Consequently, medians with interquartile ranges were used to report continuous variables, and frequencies with weighted percentages were used for categorical variables. Weighted Cox proportional hazards models were used to examine the association between NPAR and all‑cause and cardiovascular mortality.

Four sequential models were constructed. Model 1: Adjusted for NPAR and survey cycle (year). Model 2: Model 1 + demographics (age, sex, race/ethnicity, BMI) and socioeconomic factors (education, marital status, PIR). Model 3: Model 2 + lifestyle factors (smoking, alcohol) and comorbidities (diabetes, hypertension, CVD, cancer, insurance) and antihypertensive and antidiabetic. Model 4 (fully adjusted): Model 2 + lifestyle factors, comorbidities, laboratory parameters (systolic BP, diastolic BP, total cholesterol, LDL cholesterol, fasting glucose, HbA1c, eGFR), and medications (ACEI/ARBs, β-blockers, CCBs, diuretics, statins, antiplatelet agents, antidiabetic agents, urate-lowering therapy), as well as rheumatic disease. Antihypertensive medication was not included as a separate variable in Model 4 because its components (ACEI/ARBs, β-blockers, CCBs, and diuretics) were already individually modeled. To prevent mathematical coupling, albumin, neutrophil percentage, serum uric acid, and serum creatinine were excluded from all multivariable models. Notably, diuretic use was modeled as a separate binary variable given its direct influence on uric acid levels. Rheumatic diseases (rheumatoid arthritis and psoriatic arthritis) were also included as a binary variable, with gout patients excluded to avoid overlap with the study population. In the HUA subgroup, urate‑lowering therapy was excluded from Model 4 due to zero prevalence; in the gout subgroup, rheumatic disease was excluded due to insufficient events for stable estimation.

To evaluate the predictive accuracy of multivariable-adjusted NPAR for mortality outcomes in the cohort with HUA and gout, (area under the curve) AUC was utilized. The Kaplan-Meier (KM) survival curve was employed to represent censored data and to illustrate differential survival patterns across various quartiles of NPAR. Stratified Kaplan–Meier survival curves were additionally constructed separately for asymptomatic HUA and gout subgroups to visualize NPAR quartile-specific survival trajectories (Figs. S6, S7). Participants with missing data on any covariate were excluded from the respective multivariable models (complete case analysis). Missing data rates for core demographic and lifestyle variables were 0%. For laboratory variables added in Model 4, missing rates ranged from 0.3% (HbA1c) to 18.4% (fasting glucose), with 2,683 of 3,659 HUA participants and 633 of 685 gout participants having complete data for the fully adjusted model. To account for the competing risk of non‑cardiovascular death, cause‑specific Cox models were fitted via the data duplication approach with event‑type stratification, incorporating NHANES survey weights. The Fine‑Gray subdistribution hazard model was approximated using this approach [30, 31] to estimate subdistribution hazard ratios (sHR) for CVD mortality, with non‑CVD death treated as the competing event. Cumulative incidence functions were estimated by the Aalen‑Johansen method stratified by NPAR quartiles. Results are presented in Figs. S9 and S10.We applied restricted cubic spline (RCS) transformations to study the dose-response association between NPAR and both all-cause and cardiovascular mortality in patients suffering from HUA and gout. Subgroup analyses were conducted according to age (18–39, 40–57, 58–70, ≥ 71 years), gender (male or female), level of education achieved (below high school, college, or above), marital status (never married, married/living with partner, or widowed/divorced/separated), race/ethnicity (Mexican American, Other Hispanic, non-Hispanic White, non-Hispanic Black, or other races), and PIR (below 1.3, from 1.3 to 3.5, above 3.5). Further subgroup categories were based on BMI (< 30 kg/m², ≥ 30 kg/m²), smoking habits (never, former, current), alcohol use (yes/no), and medical history including hypertension, cancer, diabetes, and CVD (all yes/no). To assess the interaction between various subgroups, the likelihood ratio test was employed.

Furthermore, to evaluate whether the prognostic utility of NPAR differed between clinical phenotypes, we conducted stratified analyses by gout status. All primary analyses–-including Kaplan-Meier survival curves, multivariable Cox proportional hazards regression by NPAR quartiles, and restricted cubic spline modeling–-were repeated separately in: (i) participants with asymptomatic HUA only (without physician-diagnosed gout, *n* = 3,659) and (ii) participants with gout (*n* = 685). We additionally performed a subgroup analysis directly comparing participants with HUA only (without physician-diagnosed gout) to those with gout. Cox proportional hazards models were used to estimate hazard ratios (HRs) and 95% confidence intervals (CIs) for all-cause mortality, with progressive adjustment for confounders. This approach allowed us to evaluate whether the prognostic utility of NPAR for mortality outcomes differed between these two clinically distinct populations.

Three sensitivity analyses were conducted to confirm the robustness of the association between NPAR and mortality. To mitigate potential reverse causality, individuals who died within two years post‑interview were excluded (Table S2). Subsequently, the analysis was restricted to participants interviewed from 1999 to 2006 to evaluate temporal consistency (Table S3). Additionally, to assess whether the association was affected by different uric acid cutoff values, the main Cox analyses were repeated using three alternative UA thresholds for defining the study population: (i) the URRAH cardiovascular‑specific cutoff (≥ 333 µmol/L for both sexes), (ii) a lower URRAH cutoff (≥ 286 µmol/L), and (iii) the traditional HUA definition (≥ 420 µmol/L for men, ≥ 360 µmol/L for women) (Table S4). Given the exploratory nature of the subgroup analyses, P values for interaction were not adjusted for multiple comparisons; therefore, these results should be interpreted with caution. DecisionLinnc software (version 1.0) was utilized to carry out the statistical analysis.

## Results

### Baseline characteristics

A total of 4,344 participants were included in the study. The median age was 52 years, the median BMI was 31.03 kg/m², and the median serum uric acid level was 434.2 µmol/L. Males comprised 56.8% (*n* = 2,443) of the cohort. Non-Hispanic White participants constituted the majority (70.5%, *n* = 2,037), followed by non-Hispanic Black (12.1%, *n* = 1,014), Mexican American (5.8%, *n* = 557), Other Hispanic (4.5%, *n* = 309), and other races (7.1%, *n* = 427). More than half of the participants had at least a high school education (56.4%). Current and former smokers accounted for 17.2% and 31.2%, respectively. The most prevalent comorbidities were diabetes mellitus (20.5%), cancer (13.0%), and cardiovascular disease (12.0%). Health insurance coverage was 85.0%. The median NPAR was 13.63.

Over a median follow-up of 99 months, 887 all-cause deaths and 268 cardiovascular deaths occurred. Non-survivors differed markedly from survivors: they were older (median age 71 vs. 48 years), had a lower proportion of males (53.4% vs. 57.4%), were more likely to be non-Hispanic White (76.6% vs. 69.4%), had lower educational attainment, were more frequently widowed, divorced, or separated (40.0% vs. 18.4%), and had a higher burden of comorbidities, including diabetes (36.4% vs. 17.6%), hypertension (37.2% vs. 20.5%), cardiovascular disease (30.4% vs. 8.8%), and cancer (23.2% vs. 11.2%). Non-survivors were also less likely to be never-smokers (39.1% vs. 53.8%), had lower socioeconomic status, and exhibited higher NPAR values (*P* < 0.001 for all comparisons; Table 1). The study population was further classified into asymptomatic hyperuricemia (*n* = 3,659) and gout (*n* = 685) subgroups; baseline characteristics of these subgroups are compared in Table 2.

**Table 1:** Baseline characteristics of adults with hyperuricemia and gout. Weighted data are presented as median (IQR) for continuous variables and n (%) for categorical variables

Variables	Total (*n* = 4,344)	Survivors (*n* = 3,457)	Non-survivors (*n* = 887)	*P*-value
Follow-up time, months	99.00 (51.00–150.00)	104.00 (54.00–155.00)	72.00 (35.00–117.00)	< 0.001
Age, years	52.00 (36.00–65.00)	48.00 (33.00–61.00)	71.00 (59.00–80.00)	< 0.001
Male, n (%)	2,443 (56.8)	1,946 (57.4)	497 (53.4)	0.100
Race, n (%)				< 0.001
Mexican American	557 (5.8)	477 (6.3)	80 (3.0)	
Other Hispanic	309 (4.5)	266 (4.6)	43 (3.5)	
Non-Hispanic White	2,037 (70.5)	1,505 (69.4)	532 (76.6)	
Non-Hispanic Black	1,014 (12.1)	805 (11.8)	209 (14.1)	
Other race	427 (7.1)	404 (7.9)	23 (2.7)	
BMI, n (%)				< 0.001
Overweight	1,341 (30.1)	1,003 (29.1)	338 (35.6)	
Obese	2,365 (56.5)	1,986 (58.5)	379 (45.1)	
Education level, n (%)				< 0.001
≤High school	2,230 (43.6)	1,653 (40.8)	577 (59.2)	
College	1,279 (32.0)	1,070 (32.7)	209 (28.5)	
College	835 (24.3)	734 (26.5)	101 (12.2)	
Marital status, n (%)				< 0.001
Married/living with partner	2,523 (62.5)	2,069 (64.0)	454 (54.3)	
Widowed/divorced/separated	1,139 (21.7)	752 (18.4)	387 (40.0)	
Never married	682 (15.8)	636 (17.6)	46 (5.7)	
PIR, n (%)				< 0.001
<1.3	1,246 (20.5)	951 (19.2)	295 (27.8)	
1.3–3.5	1,656 (36.8)	1,270 (35.3)	386 (45.5)	
>3.5	1,442 (42.7)	1,236 (45.5)	206 (26.8)	
Smoking, n (%)				< 0.001
Never	2,252 (51.6)	1,900 (53.8)	352 (39.1)	
Ever	1,348 (31.2)	958 (29.1)	390 (43.1)	
Now	744 (17.2)	599 (17.1)	145 (17.8)	
Alcohol use, n (%)	3,443 (82.3)	2,845 (84.4)	598 (70.4)	< 0.001
Insurance status, n (%)	3,625 (85.0)	2,807 (83.9)	818 (91.2)	< 0.001
Hypertension, n (%)	1,134 (23.0)	778 (20.5)	356 (37.2)	< 0.001
Diabetes, n (%)	1,129 (20.5)	794 (17.6)	335 (36.4)	< 0.001
CVD, n (%)	680 (12.0)	392 (8.8)	288 (30.4)	< 0.001
Cancer, n (%)	597 (13.0)	392 (11.2)	205 (23.2)	< 0.001
Rheumatic disease, n (%)	108 (2.0)	88 (1.9)	20 (2.5)	0.311
Gout, n (%)	685 (13.9)	534 (13.5)	151 (16.2)	0.122
Uric acid, µmol/L	434.20 (392.60–469.90)	434.20 (392.60–469.90)	446.10 (404.50–493.70)	< 0.001
NPAR	13.63 (12.05–15.51)	13.45 (11.90–15.28)	14.93 (13.02–16.93)	< 0.001
eGFR, mL/min/1.73 m²	87.32 (67.65–102.37)	89.39 (72.31–104.90)	68.06 (50.12–89.39)	< 0.001
Total cholesterol, mg/dL	195.00 (167.00–224.00)	195.00 (169.00–224.00)	191.00 (160.00–222.00)	0.011
LDL cholesterol, mg/dL	113.00 (94.00–138.00)	115.00 (94.00–138.00)	108.00 (88.00–133.00)	< 0.001
Fasting glucose, mmol/L	5.72 (5.27–6.55)	5.72 (5.27–6.44)	5.83 (5.27–7.11)	0.003
HbA1c, %	5.50 (5.20–5.90)	5.50 (5.20–5.90)	5.70 (5.40–6.20)	< 0.001
Mean SBP, mmHg	123.33 (114.67–136.00)	122.67 (114.00–134.00)	131.33 (118.67–149.33)	< 0.001
Mean DBP, mmHg	72.00 (64.00–80.00)	72.67 (65.33–80.00)	68.00 (58.00–76.67)	< 0.001
ACEI/ARB, n (%)	1,419 (30.0)	1,017 (27.3)	402 (45.4)	< 0.001
β-blocker, n (%)	1,001 (19.8)	643 (16.5)	358 (38.4)	< 0.001
CCB, n (%)	643 (11.1)	430 (9.2)	213 (22.1)	< 0.001
Diuretics, n (%)	1,324 (26.2)	865 (21.9)	459 (50.5)	< 0.001
Statin, n (%)	1,091 (22.5)	786 (20.2)	305 (35.4)	0.003
Antiplatelet, n (%)	200 (3.5)	110 (2.3)	90 (9.9)	< 0.001
Antidiabetic, n (%)	715 (13.3)	501 (11.5)	214 (23.7)	< 0.001
Urate-lowering therapy, n (%)	209 (4.6)	156 (4.4)	53 (6.0)	0.076

Data are presented as median (Q1–Q3) for continuous variables and n (%) for categorical variables

*ACEI/ARB* Angiotensin-converting enzyme inhibitor/angiotensin receptor blocker, *CCB* Calcium channel blocker, *CVD* Cardiovascular disease, *DBP* Diastolic blood pressure, *eGFR* estimated glomerular filtration rate, *NPAR* Neutrophil percentage-to-albumin ratio, *PIR* Poverty-income ratio, *SBP* Systolic blood pressure

P-values for continuous variables were calculated using the Wilcoxon rank-sum test; P-values for categorical variables were calculated using the chi-square test. BMI categories: Overweight = 25.0–29.9 kg/m², Obese = ≥ 30.0 kg/m²

**Table 2:** Baseline characteristics of study participants stratified by asymptomatic hyperuricemia and gout. Data are presented as median (IQR) for continuous variables and *n* (%) for categorical variables

Variables	Total (*n* = 4,344)	HUA only (*n* = 3,659)	Gout (*n* = 685)	P-value
Demographics				
Age, years	52.00 (36.00–65.00)	49.00 (33.00–63.00)	62.00 (52.00–71.00)	< 0.001
Male	2,443 (56.8)	1,972 (55.5)	471 (65.4)	0.002
Race/Ethnicity				0.157
Mexican American	557 (5.8)	499 (6.0)	58 (4.4)	
Other Hispanic	309 (4.5)	262 (4.6)	47 (3.6)	
Non-Hispanic White	2,037 (70.5)	1,697 (70.3)	340 (72.0)	
Non-Hispanic Black	1,014 (12.1)	859 (12.3)	155 (11.4)	
Other	427 (7.1)	342 (6.8)	85 (8.6)	
Education				0.407
Less than high school	2,230 (43.6)	1,883 (44.1)	347 (40.9)	
High school or equivalent	1,279 (32.0)	1,084 (32.0)	195 (32.1)	
College or above	835 (24.3)	692 (23.9)	143 (27.1)	
Marital status				< 0.001
Married/Living with partner	2,523 (62.5)	2,072 (61.2)	451 (70.4)	
Widowed/Divorced/Separated	1,139 (21.7)	951 (21.5)	188 (22.8)	
Never married	682 (15.8)	636 (17.3)	46 (6.8)	
Poverty-income ratio (PIR)				0.307
<1.3	1,246 (20.5)	1,038 (20.3)	208 (22.0)	
1.3–3.5	1,656 (36.8)	1,409 (37.4)	247 (33.1)	
>3.5	1,442 (42.7)	1,212 (42.3)	230 (44.9)	
Clinical measurements				
BMI, kg/m²	31.03 (27.19–36.34)	30.98 (27.20–36.41)	31.20 (27.00–35.90)	0.693
Systolic BP, mmHg	123.33 (114.67–136.00)	122.67 (114.67–134.67)	128.00 (115.33–143.33)	< 0.001
Diastolic BP, mmHg	72.00 (64.00–80.00)	72.67 (64.67–80.00)	70.00 (62.00–77.33)	< 0.001
Serum uric acid, µmol/L	434.20 (392.60–469.90)	440.20 (404.50–475.80)	392.60 (321.20–458.00)	< 0.001
Serum creatinine, mg/dL	0.92 (0.80–1.10)	0.91 (0.80–1.10)	0.97 (0.82–1.17)	< 0.001
eGFR, mL/min/1.73 m²	87.32 (67.65–102.37)	88.98 (69.86–103.51)	78.27 (62.34–92.99)	< 0.001
Total cholesterol, mg/dL	195.00 (167.00–224.00)	196.00 (169.00–225.00)	184.00 (155.00–212.00)	< 0.001
LDL cholesterol, mg/dL	113.00 (94.00–138.00)	115.00 (94.00–138.00)	104.00 (79.00–133.00)	< 0.001
Fasting glucose, mmol/L	5.72 (5.27–6.55)	5.66 (5.27–6.49)	6.00 (5.50–6.94)	< 0.001
HbA1c,	5.50 (5.20–5.90)	5.50 (5.20–5.90)	5.70 (5.40–6.20)	< 0.001
NPAR	13.63 (12.05–15.51)	13.56 (11.98–15.37)	14.24 (12.45–16.32)	< 0.001
BMI category				0.519
Overweight	1,341 (30.1)	1,126 (30.1)	215 (29.9)	
Obese	2,365 (56.5)	2,003 (56.5)	362 (56.3)	
Lifestyle factors				
Smoking status				< 0.001
Never	2,252 (51.6)	1,961 (52.7)	291 (44.7)	
Former	1,348 (31.2)	1,062 (29.4)	286 (42.6)	
Current	744 (17.2)	636 (17.9)	108 (12.8)	
Alcohol consumption	3,443 (82.3)	2,941 (83.2)	502 (76.5)	0.002
Comorbidities				
Diabetes mellitus	1,129 (20.5)	839 (18.0)	290 (35.8)	< 0.001
Hypertension	1,134 (23.0)	882 (21.6)	252 (32.2)	< 0.001
Cardiovascular disease	680 (12.0)	482 (10.3)	198 (22.8)	< 0.001
Cancer	597 (13.0)	465 (11.4)	132 (22.6)	< 0.001
Rheumatic disease	108 (2.0)	108 (2.3)	0 (0.0)	0.004
Health insurance	3,625 (85.0)	2,997 (84.0)	628 (91.6)	0.003
Medications				
Antihypertensive	2,275 (46.9)	1,788 (43.6)	487 (67.7)	< 0.001
ACE inhibitor/ARB	1,419 (30.0)	1,081 (26.9)	338 (49.2)	< 0.001
Beta-blocker	1,001 (19.8)	748 (17.9)	253 (31.6)	< 0.001
Calcium channel blocker	643 (11.1)	484 (10.0)	159 (18.3)	< 0.001
Diuretics	1,324 (26.2)	1,080 (25.2)	244 (32.5)	0.01
Statin	1,091 (22.5)	789 (19.2)	302 (42.9)	< 0.001
Antiplatelet	200 (3.5)	149 (3.0)	51 (6.3)	0.001
Antidiabetic	715 (13.3)	512 (11.5)	203 (24.1)	< 0.001
Urate-lowering therapy	209 (4.6)	0 (0.0)	209 (33.0)	< 0.001
Outcomes				
Follow-up time, months	99.00 (51.00–150.00)	104.00 (55.00–160.00)	67.00 (32.00–107.00)	< 0.001
All-cause death	887 (15.1)	736 (14.7)	151 (17.5)	0.122
Cardiovascular death	268 (4.6)	228 (4.6)	40 (4.3)	0.721

### Association between NPAR and mortality

**Fig. 2:**
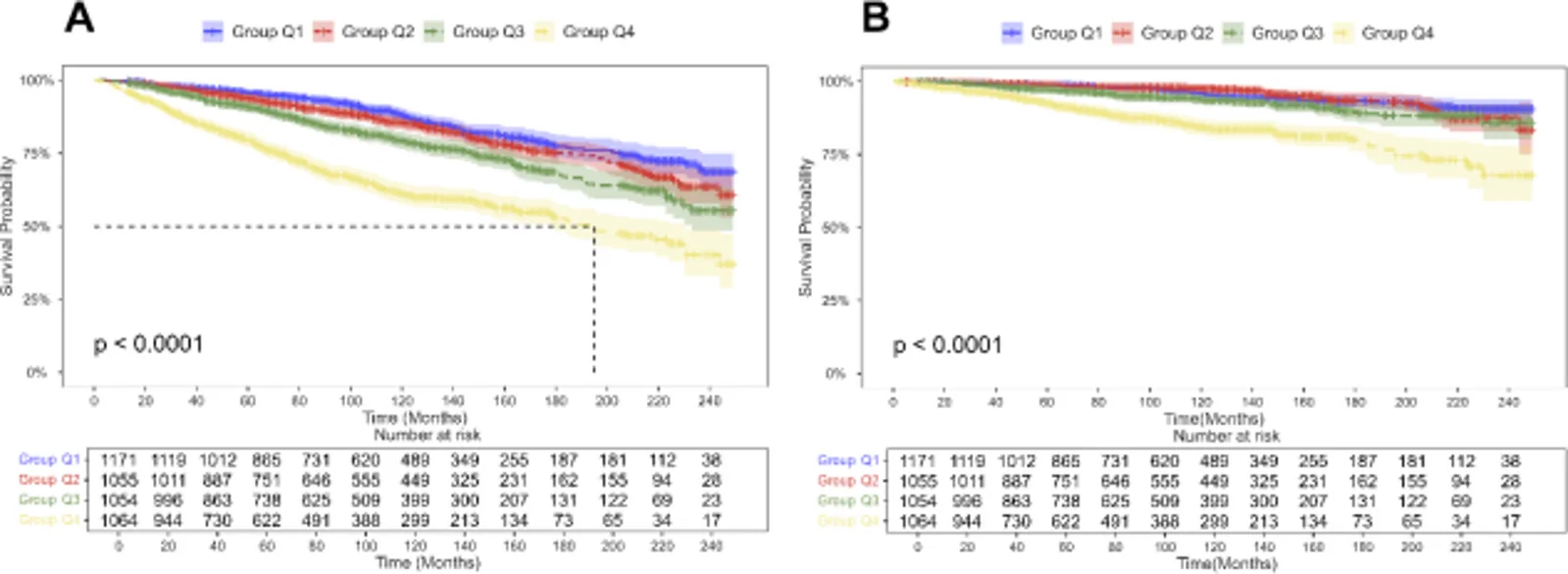
Kaplan–Meier curves illustrate the survival trends of adults with HUA and gout across various quartiles of NPAR indices. **A** represents the all-cause mortality for these adults at different NPAR quartile levels; **B** indicates the cardiovascular death rates among adults with HUA and gout across various quartile levels of NPAR. NPAR, Neutrophil percentage to albumin ratio; Q, quartile; HUA, hyperuricemia

Kaplan–Meier survival curves for all-cause and cardiovascular mortality, stratified by NPAR quartiles, are presented in Fig. 2. Both outcomes showed statistically significant differences across quartiles (log-rank *P* < 0.001). For all-cause mortality, survival curves diverged markedly during follow-up, with estimated survival probabilities of approximately 70% in Q1 and 35% in Q4 at 20 years. Cardiovascular mortality showed a more gradual but consistent divergence across quartiles.

**Fig. 3:**
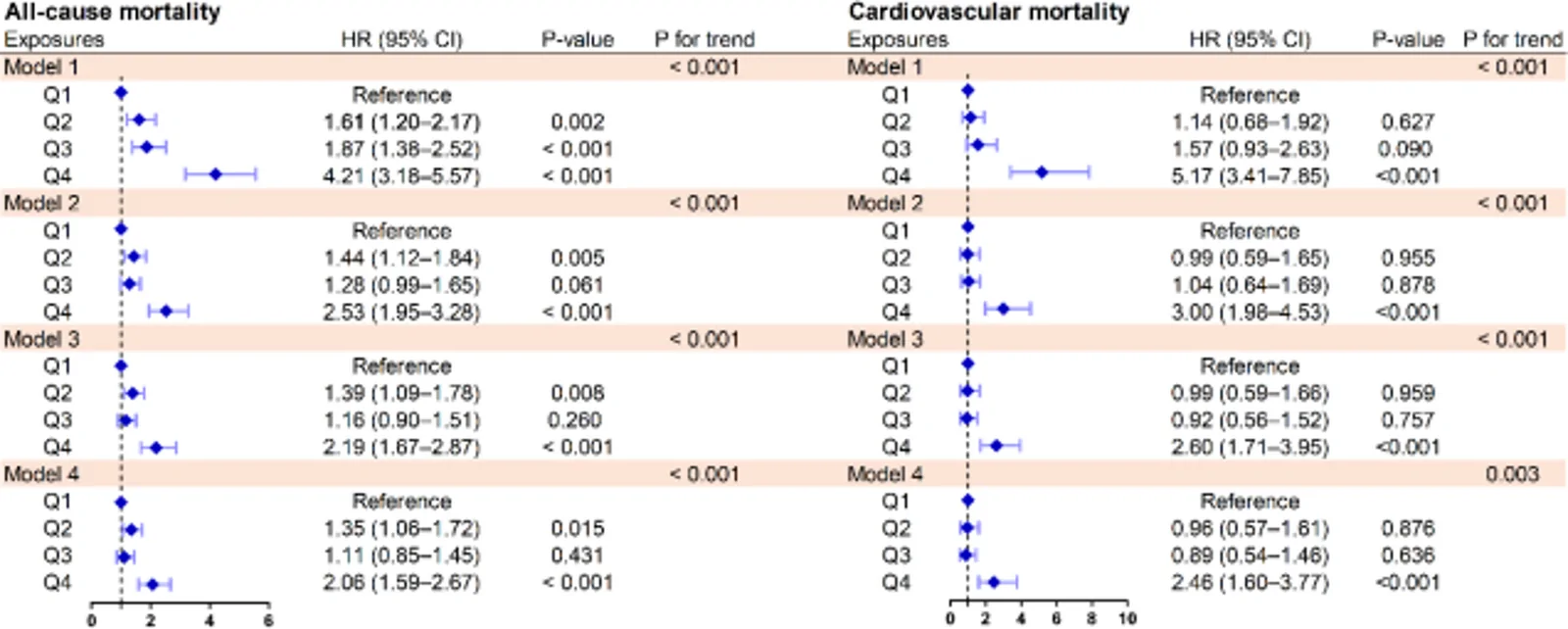
Forest plots showing the association between NPAR quartiles and all-cause mortality and cardiovascular mortality in adults with hyperuricemia and gout. NPAR, neutrophil percentage-to-albumin ratio; Q, quartile. Model 1: Adjusted for NPAR and survey cycle (year). Model 2: Model 1 + demographics (age, sex, race/ethnicity, BMI) and socioeconomic factors (education, marital status, PIR). Model 3: Model 2 + lifestyle factors (smoking, alcohol) and comorbidities (diabetes, hypertension, CVD, cancer, insurance). Model 4 (fully adjusted): Model 2 + lifestyle factors, comorbidities, laboratory parameters (systolic BP, diastolic BP, total cholesterol, LDL cholesterol, fasting glucose, HbA1c, eGFR), and medications (ACEI/ARBs, β-blockers, CCBs, diuretics, statins, antiplatelet agents, antidiabetic agents, urate-lowering therapy), as well as rheumatic disease

In the fully adjusted model (Model 4), the highest NPAR quartile (Q4) was significantly associated with increased mortality compared with Q1. Among participants with asymptomatic HUA, the adjusted hazard ratios were 2.08 (95% CI: 1.56–2.78; *P* < 0.001) for all-cause mortality and 2.24 (95% CI: 1.41–3.56; *P* = 0.001) for cardiovascular mortality (Table 3) (Fig. 3).

**Table 3:** Stratified association between NPAR and all‑cause and cardiovascular mortality by clinical subgroup

Outcome	Subgroup	Model	Q2	Q3	Q4	*P* for trend*
CVD mortality	**Gout**	Model 1	0.97 (0.19–4.90)	0.88 (0.23–3.35)	6.24 (1.78–21.83)	< 0.001
			*P* = 0.973	*P* = 0.852	***P*** ** = 0.004**	
		Model 2	1.01 (0.24–4.33)	0.71 (0.19–2.66)	5.12 (1.53–17.11)	< 0.001
			*P* = 0.991	*P* = 0.610	***P*** ** = 0.008**	
		Model 3	0.73 (0.14–3.67)	0.44 (0.08–2.26)	5.01 (1.14–21.97)	< 0.001
			*P* = 0.699	*P* = 0.322	***P*** ** = 0.033**	
		Model 4	0.53 (0.13–2.25)	0.23 (0.05–1.13)	3.51 (0.95–13.01)	0.002
			*P* = 0.392	*P* = 0.071	*P* = 0.060	
	**Asymptomatic HUA**	Model 1	1.14 (0.64–2.03)	1.65 (0.94–2.89)	4.94 (3.12–7.81)	< 0.001
			*P* = 0.647	*P* = 0.080	***P*** ** < 0.001**	
		Model 2	0.96 (0.55–1.65)	1.10 (0.66–1.85)	2.76 (1.78–4.26)	< 0.001
			*P* = 0.871	*P* = 0.711	***P*** ** < 0.001**	
		Model 3	0.96 (0.56–1.65)	1.00 (0.59–1.70)	2.43 (1.55–3.80)	< 0.001
			*P* = 0.877	*P* = 0.997	***P*** ** < 0.001**	
		Model 4	0.94 (0.54–1.65)	0.94 (0.56–1.60)	2.24 (1.41–3.56)	< 0.001
			*P* = 0.827	*P* = 0.826	***P*** ** = 0.001**	
All-cause mortality	**Gout**	Model 1	1.38 (0.69–2.75)	1.88 (0.97–3.64)	3.77 (2.09–6.80)	< 0.001
			*P* = 0.360	*P* = 0.060	***P*** ** < 0.001**	
		Model 2	1.42 (0.76–2.65)	1.33 (0.68–2.60)	2.54 (1.42–4.53)	< 0.001
			*P* = 0.269	*P* = 0.403	***P*** ** = 0.002**	
		Model 3	1.51 (0.79–2.90)	1.15 (0.62–2.13)	2.34 (1.32–4.14)	< 0.001
			*P* = 0.211	*P* = 0.663	***P*** ** = 0.004**	
		Model 4	1.32 (0.70–2.50)	0.96 (0.50–1.82)	2.14 (1.09–4.23)	0.002
			*P* = 0.391	*P* = 0.895	*P* = 0.028	
	**Asymptomatic HUA**	Model 1	1.62 (1.17–2.24)	1.77 (1.26–2.50)	4.17 (3.07–5.66)	< 0.001
			*P* = 0.004	*P* = 0.001	***P*** ** < 0.001**	
		Model 2	1.45 (1.10–1.92)	1.26 (0.94–1.69)	2.54 (1.91–3.37)	< 0.001
			***P*** ** = 0.009**	*P* = 0.128	***P*** ** < 0.001**	
		Model 3	1.44 (1.10–1.88)	1.17 (0.87–1.57)	2.26 (1.68–3.03)	< 0.001
			***P*** ** = 0.009**	*P* = 0.303	***P*** ** < 0.001**	
		Model 4	1.38 (1.06–1.81)	1.10 (0.82–1.48)	2.08 (1.56–2.78)	< 0.001
			***P*** ** = 0.018**	*P* = 0.525	***P*** ** < 0.001**	

Bold indicates statistically significant hazard ratios (*P* < 0.05)

Reference: NPAR Q1. * *P* for trend derived from NPAR as continuous variable (per 1‑SD increment) in the same model

Model covariates:

M1: NPAR + year

M2: M1 + age, sex, race, BMI, education, marital status, PIR

M3: M2 + smoking, alcohol, diabetes, hypertension, CVD, cancer, insurance, antihypertensive, antidiabetic

M4: M2 + smoking, alcohol, diabetes, hypertension, CVD, cancer, insurance, antidiabetic, SBP, DBP, TC, LDL‑C, glucose, HbA1c, eGFR, ACEI/ARB, β‑blocker, CCB, diuretics, statin, antiplatelet; HUA grouP additionally adjusted for rheumatic disease; Gout grouP additionally adjusted for urate‑lowering therapy

### Predictive value of NPAR

**Fig. 4:**
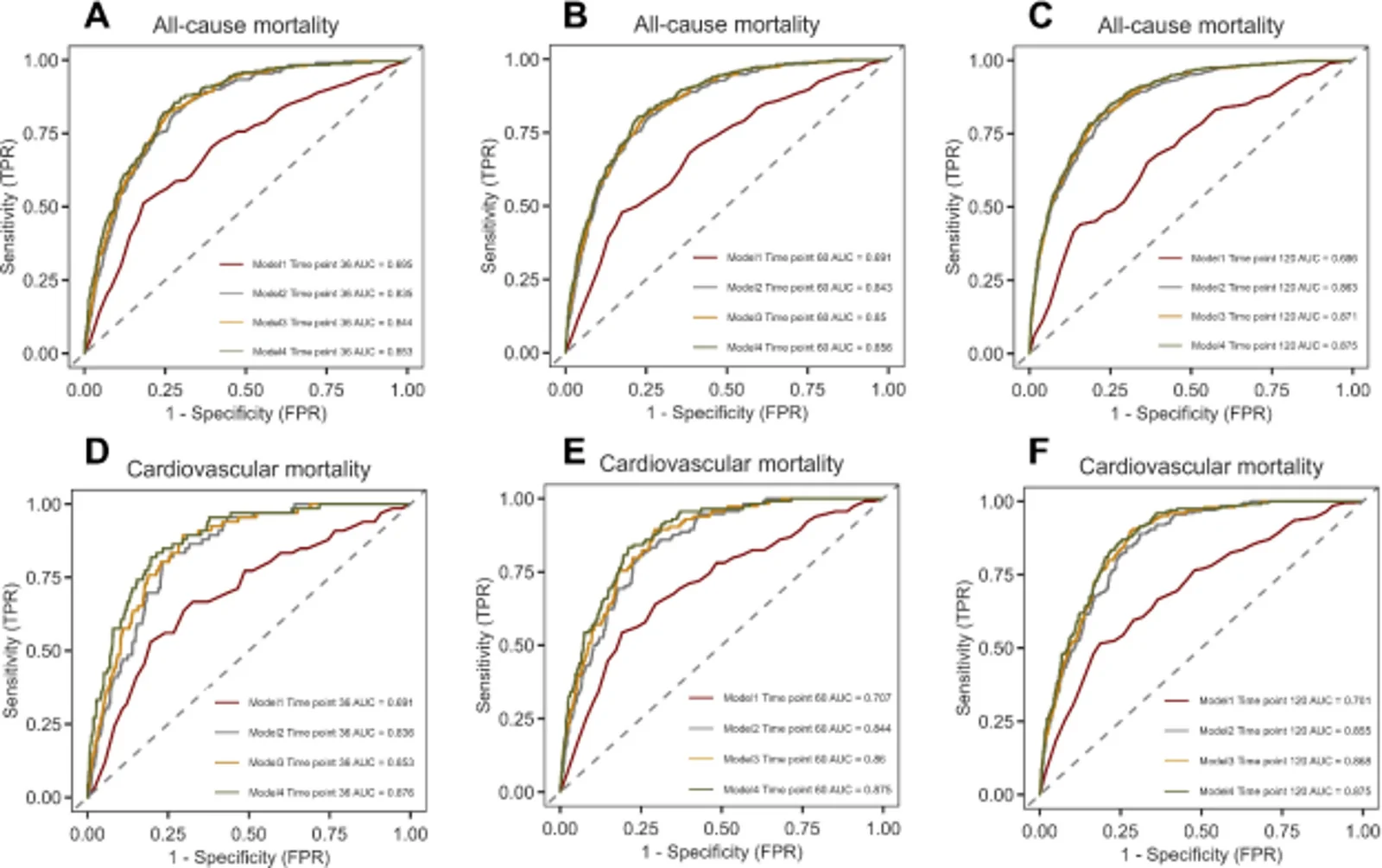
The ROC curves of NPAR for predicting outcomes in populations with HUA and gout. **A**–**C** ROC curve analysis of NPAR for all-cause mortality at 36, 60, and 120 months, respectively. **D**–**F** ROC curve analysis of NPAR for cardiovascular mortality at 36, 60, and 120 months, respectively. Model 1 was adjusted for NPAR and survey year. Model 2 was additionally adjusted for age, gender, race, BMI, education level, marital status, and PIR. Model 3 was additionally adjusted for smoking, alcohol consumption, diabetes, hypertension, cardiovascular disease, cancer, insurance coverage, antihypertensive medication, and antidiabetic medication. Model 4 was adjusted for Model 2 variables plus smoking, alcohol consumption, diabetes, hypertension, cardiovascular disease, cancer, insurance coverage, antidiabetic medication, mean systolic blood pressure, mean diastolic blood pressure, total cholesterol, LDL cholesterol, glucose, HbA1c, eGFR, ACE inhibitors/ARBs, beta-blockers, calcium channel blockers, diuretics, statins, antiplatelet agents, urate-lowering therapy, rheumatic disease, and survey year

Time-dependent ROC curve analysis demonstrated strong predictive performance for NPAR across 3-, 5-, and 10-year horizons (Fig. 4). In Model 4, the area under the curve (AUC) for all-cause mortality was 0.853, 0.856, and 0.875 at 3, 5, and 10 years, respectively; corresponding values for cardiovascular mortality were 0.876, 0.875, and 0.875.

To assess the incremental prognostic value of NPAR beyond traditional risk factors, we evaluated the C-index, net reclassification improvement (NRI), integrated discrimination improvement (IDI), and median improvement at 5 years (Supplementary Table S5). Adding NPAR to the base model (which included demographics, lifestyle factors, comorbidities, and health insurance) improved the C-index by 0.010 for all-cause mortality and 0.016 for cardiovascular mortality in the full cohort. The IDI was 0.034 (*P* < 0.001) and 0.032 (*P* < 0.001), the NRI was 0.197 (*P* < 0.001) and 0.311 (*P* < 0.001), and the median improvement was 0.015 (*P* < 0.001) and 0.008 (*P* < 0.001), for all-cause and cardiovascular mortality, respectively.

Similar patterns were observed in the asymptomatic HUA subgroup, with significant improvements in discrimination and reclassification (all *P* < 0.001). In the gout subgroup, NPAR conferred even greater incremental value for all-cause mortality (ΔC-index: 0.029; IDI: 0.066, *P* < 0.001; NRI: 0.227, *P* < 0.001), though the median improvement did not reach significance (0.025, *P* = 0.06). For cardiovascular mortality in the gout subgroup, the ΔC-index was 0.025, the IDI was 0.083 (*P* = 0.04), and the NRI was 0.377 (*P* = 0.04); the median improvement was 0.039 (*P* = 0.159).

These findings indicate that NPAR provides significant incremental prognostic information beyond established risk factors across the full cohort and most clinical subgroups.

### Dose–response relationship between NPAR and mortality

**Fig. 5:**
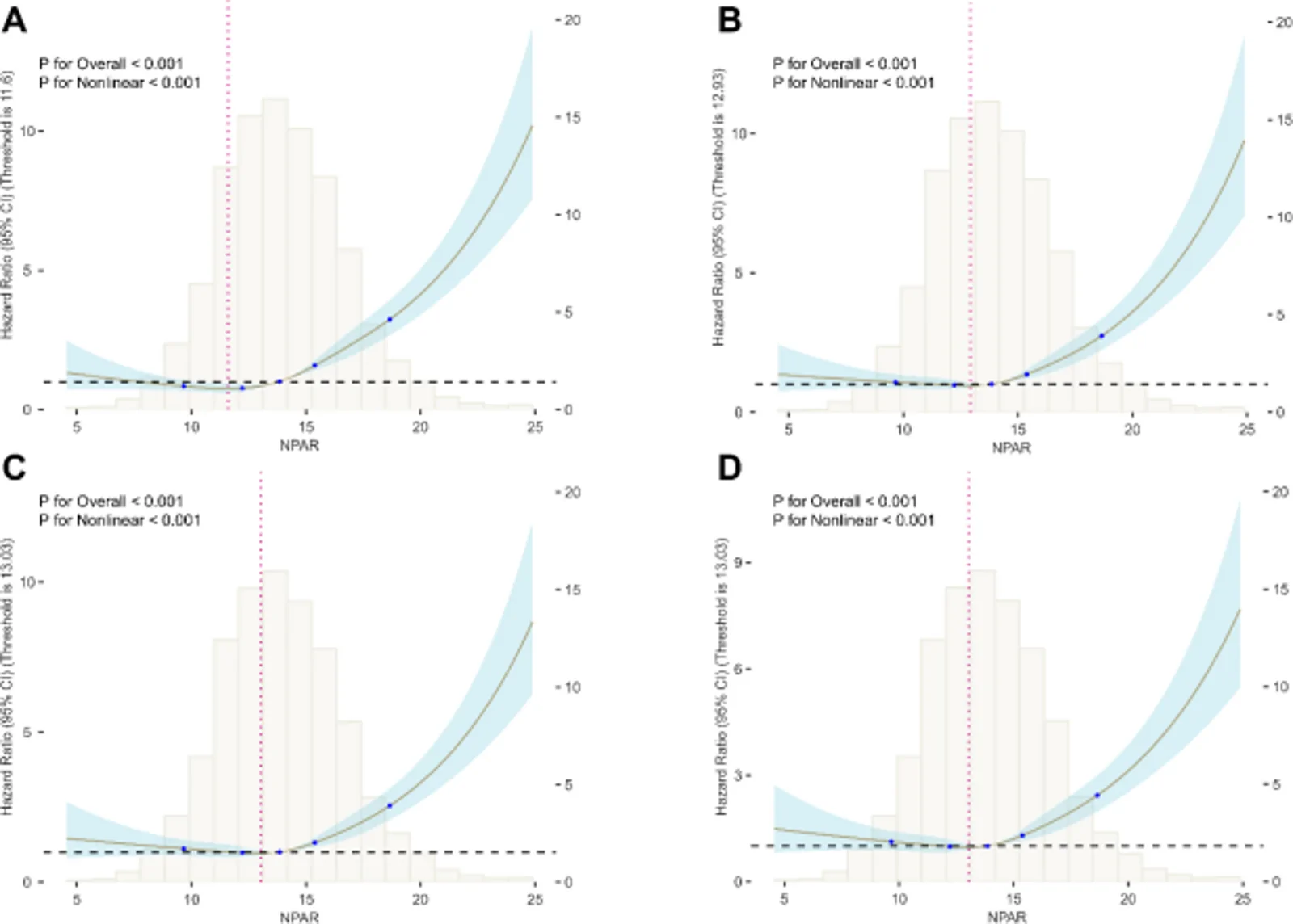
Restricted cubic spline (RCS) curves showing the dose-response relationship between NPAR and mortality risk. **A**–**D** All-cause mortality in Model 1 through Model 4, respectively. The red dotted line indicates the inflection point where HR = 1. P for Overall and P for Nonlinear were calculated using likelihood ratio tests. The histogram in the background represents the distribution of NPAR in the study population

**Fig. 6:**
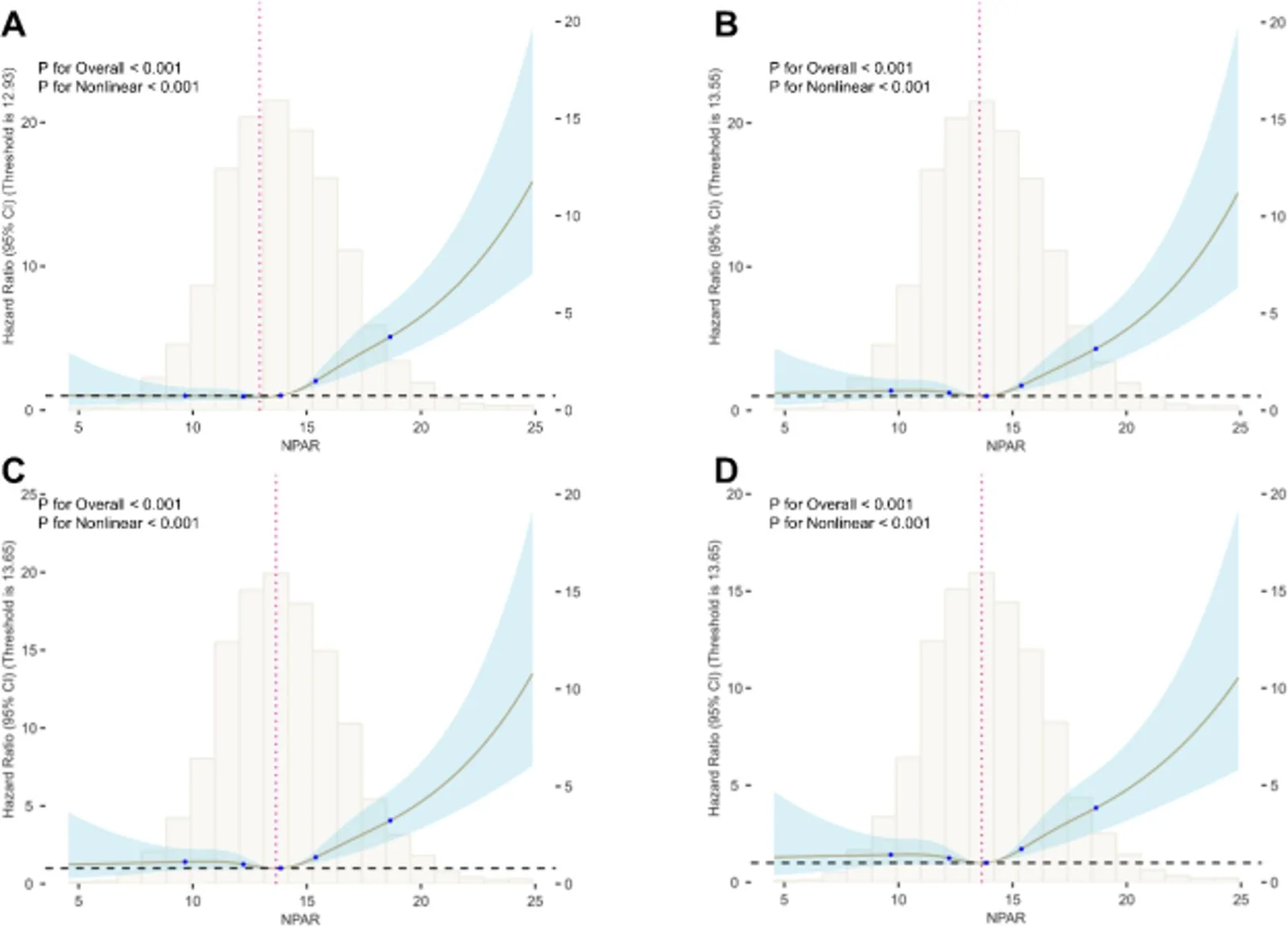
Restricted cubic spline (RCS) curves showing the dose-response relationship between NPAR and mortality risk. **A**–**D** Cardiovascular mortality in Model 1 through Model 4, respectively. The red dotted line indicates the inflection point where HR = 1. P for Overall and P for Nonlinear were calculated using likelihood ratio tests. The histogram in the background represents the distribution of NPAR in the study population

Restricted cubic spline (RCS) analyses revealed significant non-linear associations between NPAR and both all-cause mortality (overall *P* < 0.001; *P* for non-linearity < 0.001) and cardiovascular mortality (overall *P* < 0.001; *P* for non-linearity = 0.001) (Figs. 5 and 6). In the crude model (Model 1), the inflection points were identified at NPAR = 11.6 for all-cause mortality and NPAR = 12.93 for cardiovascular mortality.

We further examined these relationships stratified by clinical phenotype (Figs. S4 and S5). In the asymptomatic HUA subgroup, the fully adjusted Model 4 showed a shallow J-shaped pattern for all-cause mortality: no significant excess risk was observed at low NPAR levels (nadir HR = 0.97, 95% CI: 0.91–1.03 at NPAR ≈ 12.6), whereas higher NPAR was significantly associated with increased mortality (peak HR = 2.12, 95% CI: 1.77–2.53 at NPAR ≈ 18.7; *P* for non-linearity = 0.0001). A similar J-shaped pattern emerged for cardiovascular mortality (nadir HR = 0.97, 95% CI: 0.89–1.06; peak HR = 2.53, 95% CI: 1.95–3.28; *P* for non-linearity = 0.004).

In the gout subgroup, higher NPAR remained significantly associated with increased all-cause mortality (peak HR = 2.99, 95% CI: 2.09–4.30 at NPAR ≈ 19.7; *P* for non-linearity = 0.009). For cardiovascular mortality, the RCS curve showed a monotonic, approximately linear increase (peak HR = 4.95, 95% CI: 2.63–9.32), though this estimate reflects the risk at the extreme upper tail of the NPAR distribution and should be interpreted alongside the quartile-based HR of 3.51 (95% CI: 0.95–13.01), which represents the average risk across Q4.

### Subgroup, stratified, and sensitivity analyses

#### Subgroup analyses

The association between NPAR and all-cause mortality was consistently positive across most demographic and clinical subgroups (Fig. S2). The prognostic value was more pronounced among males (Q4 HR = 2.41, 95% CI: 1.68–3.45) than females (Q4 HR = 1.78, 95% CI: 1.21–2.62; *P* for interaction = 0.011), and among participants with hypertension (Q4 HR = 2.15, 95% CI: 1.42–3.26) than those without (Q4 HR = 1.89, 95% CI: 1.31–2.72; *P* for interaction = 0.003). For cardiovascular mortality (Fig. S3), stronger associations were observed in older adults (≥ 60 years) and those with pre-existing CVD, though the interaction was nominally significant only for age (*P* for interaction = 0.018) and did not survive Bonferroni correction for 15 tests (adjusted threshold *P* < 0.0033). Only the hypertension interaction remained significant after Bonferroni correction, suggesting that hypertension may modify the association between NPAR and mortality. These subgroup findings should be interpreted with caution given reduced sample sizes and event numbers in certain strata, and require validation in larger cohorts.

#### Stratified analyses by gout status

To evaluate whether the prognostic utility of NPAR differed between clinical phenotypes, all primary analyses were repeated separately in participants with asymptomatic HUA only (*n* = 3,659) and those with gout (*n* = 685).

##### Kaplan–Meier survival curves

In the asymptomatic HUA subgroup (Fig. S6), Q4 exhibited significantly lower survival probabilities for both all-cause mortality (log-rank *P* < 0.001) and cardiovascular mortality (log-rank *P* < 0.001) compared with Q1, with curves diverging as early as 36 months post-enrollment. In the gout subgroup (Fig. S7), a similar pattern was observed, though separation was less pronounced for cardiovascular mortality (log-rank *P* = 0.032), reflecting the smaller event count (*n* = 40 CVD deaths).

##### Dose–response relationships

RCS analyses confirmed significant non-linear associations in both subgroups (Figs. S4, S5). In the asymptomatic HUA subgroup, Model 4 showed a shallow J-shaped pattern for all-cause mortality (*P* for non-linearity = 0.0001) and a marked elevation at high NPAR for CVD mortality (*P* for non-linearity = 0.004). In the gout subgroup, higher NPAR remained significantly associated with all-cause mortality (peak HR = 2.99, 95% CI: 2.09–4.30; *P* for non-linearity = 0.009). For CVD mortality, the RCS curve showed a monotonic increase (peak HR = 4.95, 95% CI: 2.63–9.32), though this estimate reflects the extreme upper tail risk and should be interpreted alongside the quartile-based HR of 3.51 (95% CI: 0.95–13.01).

##### Direct comparison between phenotypes

The gout group had a slightly higher crude mortality rate than the HUA-only group (23.0% vs. 21.2%). In the unadjusted model, gout was associated with higher all-cause mortality (HR = 1.83, 95% CI: 1.55–2.17, *P* < 0.001), but this association was markedly attenuated after adjustment for age, sex, and race (HR = 1.04, 95% CI: 0.88–1.24, *P* = 0.66). In the fully adjusted model, no significant difference was observed (HR = 0.91, 95% CI: 0.76–1.08, *P* = 0.29), indicating that the apparent excess mortality in gout is largely explained by demographic and clinical confounders.

#### Sensitivity analyses

Three sensitivity analyses were conducted to evaluate the robustness of the NPAR–mortality association. First, after excluding participants who died within two years of enrollment, the associations with both all-cause and cardiovascular mortality remained essentially unchanged (Supplementary Table S2). Second, restricting the analysis to participants from 1999 to 2006 yielded consistent results, although Model 4 could not be estimated due to insufficient events (Supplementary Table S3). Third, using alternative serum uric acid cutoffs—including the cardiovascular-specific threshold (≥ 333 µmol/L) and a lower threshold (≥ 286 µmol/L)—NPAR Q4 remained significantly associated with all-cause and CVD mortality in the fully adjusted model (Supplementary Table S4). These findings demonstrate that NPAR maintains prognostic significance across the spectrum of uric acid elevation, including levels below the traditional HUA threshold.

### Competing risks analysis for CVD mortality

We performed a competing risks analysis using Fine–Gray subdistribution hazard models to evaluate CVD mortality with non-CVD death as the competing event. Cumulative incidence function (CIF) curves stratified by NPAR quartile showed a clear gradient of CVD mortality risk, with Q4 exhibiting the highest cumulative incidence over 20 years of follow-up (Fig. S9).

In the Fine–Gray regression, the association between NPAR and CVD mortality remained significant across all adjustment levels (Fig. S10). In Model 1, Q4 had an sHR of 2.306 (95% CI: 1.689–3.149, *P* < 0.001) compared with Q1. After progressive adjustment, the association remained robust (Model 2: sHR = 1.910, 95% CI: 1.393–2.621; Model 3: sHR = 1.931, 95% CI: 1.404–2.655; all *P* < 0.001). In Model 4, Q4 remained independently associated with CVD mortality (sHR = 1.899, 95% CI: 1.366–2.640, *P* < 0.001), while Q2 (sHR = 0.927, 95% CI: 0.618–1.391, *P* = 0.710) and Q3 (sHR = 1.028, 95% CI: 0.692–1.529, *P* = 0.890) were not significant. The *P* for trend across quartiles was < 0.001 in all models.

In Model 4, additional variables significantly associated with CVD mortality included age, female sex, CVD history, HbA1c, diuretic use, and alcohol use (Fig. S11). Of note, sHRs from Fine–Gray models are expected to be smaller than cause-specific HRs because the former retain individuals who experience competing events in the risk set, whereas the latter censor them.

## Discussion

This study demonstrates that NPAR is an independent predictor of all-cause and cardiovascular mortality in patients with asymptomatic HUA. NPAR demonstrated a significant dose‑response relationship with mortality in both outcomes. In the asymptomatic HUA subgroup, participants in the highest NPAR quartile (Q4) exhibited a 2.08‑fold higher risk of all‑cause mortality (95% CI: 1.56–2.78, *P* < 0.001) and a 2.24‑fold higher risk of cardiovascular mortality (95% CI: 1.41–3.56, *P* = 0.001) after full adjustment, compared with Q1. In the gout subgroup, the corresponding HR was 2.14 (95% CI: 1.09–4.23, *P* = 0.028) for all‑cause mortality, while the CVD mortality association was attenuated after full adjustment (HR = 3.51, 95% CI: 0.95–13.01, *P* = 0.060), likely reflecting the limited number of CVD events (*n* = 40) in this subgroup. These associations remained stable after adjusting for conventional risk factors including BMI, diabetes, and smoking. This is the first large‑scale population‑based study to validate NPAR as a prognostic marker in patients with HUA or gout, with separate analyses by clinical phenotype. The findings contribute to understanding the role of inflammation‑nutrition imbalance in mortality risk among these populations and suggest that NPAR may serve as a straightforward and cost‑effective tool for clinical risk stratification.

An elevated NPAR is significantly correlated with increased overall and cardiovascular mortality rates in patients with HUA and gout. These observations are in agreement with past studies showing that elevated uric acid is associated with an increased risk of cardiovascular events [2, 3], consistent with the established role of uric acid in cardiovascular pathology. NPAR has similarly been linked to adverse outcomes across diverse populations [20–24]. An expanding body of evidence suggests that the chronic inflammatory state significantly influences the long-term prognosis of patients with HUA or gout. The extant body of literature predominantly underscores NPAR’s predictive utility in the context of diverse clinical conditions, with particular emphasis on its well-documented correlations with heart failure [22] and chronic kidney disease [23]. The investigation by Liu et al. demonstrated that NPAR is associated with all-cause and cardiovascular mortality in the general US adult population [15]. Nevertheless, there is a paucity of evidence concerning its applicability to populations with HUA or gout. To address this gap, the study uses a considerable sample size of 4,344 and employs multivariate adjustments to bolster the findings’ robustness. In contrast to the exclusive dependence on serum uric acid levels [14], NPAR offers a more comprehensive assessment by incorporating markers of inflammation (neutrophil percentage) and nutritional status (albumin), thus providing a holistic perspective on the pathophysiological mechanisms underlying disease progression [14]. The incremental value analyses further supported the clinical utility of NPAR. Adding NPAR to a base model of traditional risk factors improved the C‑index by 0.010 for all‑cause mortality and 0.016 for cardiovascular mortality, with significant NRI and IDI values (all *P* < 0.001). These findings indicate that NPAR captures prognostic information beyond that provided by conventional risk factors and serum uric acid alone. Furthermore, our subgroup analysis comparing patients with HUA only to those with gout revealed that after comprehensive adjustment for confounders, no significant difference in all-cause mortality was observed between the two groups (HR = 0.91, 95% CI: 0.76–1.08, *P* = 0.29; Table 3). This finding suggests that the previously reported excess mortality risk in gout patients may be largely attributable to differences in baseline demographics and comorbidities rather than to gout per se, reinforcing the notion that NPAR captures a shared inflammatory-nutritional pathway common to both conditions.

Furthermore, the competing risks analysis using the Fine-Gray model confirmed that the association between NPAR and CVD mortality remained robust even after accounting for non-CVD death as a competing event. The elevated risk associated with the highest NPAR quartile persisted across all adjustment levels (fully adjusted sHR = 1.899, 95% CI: 1.366–2.640, *P* < 0.001), with a significant dose-response trend across quartiles (P for trend < 0.001). These findings strengthen the evidence for NPAR as an independent predictor of cardiovascular outcomes in patients with HUA and gout.

The prognostic utility of NPAR may be mediated by the “chronic inflammation–endothelial injury” pathway. HUA activates the NLRP3 inflammasome [26, 32], promotes neutrophil infiltration [33], and stimulates neutrophils to release proteases particularly matrix metalloproteinase-9 (MMP-9) and reactive oxygen species (ROS), thereby accelerating vascular endothelial damage [13]. Conversely, hypoalbuminemia serves as an indicator of malnutrition or a micro-inflammatory state, consequently diminishing its antioxidant and anti-inflammatory capacities [20, 34]. These conditions collectively exacerbate oxidative stress, thereby accelerating the progression of atherosclerosis and increasing the risk of cardiovascular events. This mechanism is corroborated by experimental studies demonstrating that uric acid can downregulate nitric oxide (NO) synthesis [35], whereas albumin supplementation has been shown to enhance microcirculation [36]. Additional evidence includes: (i) animal models demonstrating that uric acid crystals directly induce myocardial fibrosis [26]; and (ii) the cardioprotective effects of colchicine and other anti-inflammatory therapies in patients with HUA [37]. In participants with hypertension, the influence of NPAR on mortality risk was significantly greater, with an interaction P-value of 0.003. The model indicated that the association between NPAR and all-cause mortality was modified by hypertension status: each 1 SD increase in NPAR was associated with an HR of 1.27 in individuals without hypertension, compared with 1.16 in those with hypertension (*P* for interaction = 0.003; Figure S2). A plausible explanation is that hypertension modifies the prognostic effect of NPAR on mortality, possibly reflecting distinct vascular inflammatory mechanisms or competing risk factors in hypertensive individuals. Angiotensin II receptor activation has been shown to promote proximal tubular growth and hypertrophy, leading to the coordinated upregulation of apical transporters including URAT1, which enhances uric acid reabsorption and perpetuates a vicious cycle of hypertension and hyperuricemia [38]. Furthermore, pathological mechanical forces associated with hypertension—including elevated hydrostatic pressure and cyclic stretch—induce endothelial dysfunction by increasing reactive oxygen species production and oxidative stress, thereby damaging the vascular endothelium [39]. These mechanistic observations align with clinical trial evidence demonstrating that urate-lowering and anti-inflammatory strategies improve cardiovascular outcomes in at-risk populations [7, 37]. These findings suggest that NPAR may serve as a biomarker to identify high-risk individuals who could benefit from enhanced antihypertensive and anti-inflammatory treatments. The predictive value of NPAR for cardiovascular mortality was more pronounced in men (per 1‑SD HR = 1.34) than in women (per 1‑SD HR = 1.24; Figure S3). Estrogen is known to lower uric acid levels by inhibiting xanthine oxidase [40], whereas men generally have a higher proportion of visceral fat, which can exacerbate inflammatory processes [41, 42]. Furthermore, the increased prevalence of smoking and drinking among men may exacerbate the risks associated with the rise in NPAR. This observation aligns with the gender disparities in CVD documented by Wang et al. [8] ; however, further research is required to establish intervention thresholds specific to each gender. Although obesity is a recognized risk factor for HUA, among participants with BMI < 30 kg/m², each 1‑SD increase in NPAR was associated with a higher mortality risk (HR = 1.27) compared with those with BMI ≥ 30 kg/m² (HR = 1.24; P for interaction = 0.101; Figure S2). This counterintuitive finding may be attributed to the “obesity paradox,” where malnutrition and inflammatory depletion are more prevalent in the low BMI group. In patients with cardiovascular disease, malnutrition has been identified as an independent predictor of poor survival (HR 2.64, 95% CI: 2.24–3.12), and the obesity paradox appears to be largely driven by nutritional status, with overweight and obese individuals demonstrating substantially lower mortality in the presence of malnutrition. Conversely, underweight patients consistently exhibit the highest mortality rates, which remain unchanged despite improvements in other BMI groups over time [43]. Additionally, the low albumin levels observed in patients with sarcopenia may obscure the true extent of the inflammatory burden [44], with NPAR demonstrating its relevance. Furthermore, the choice of uric acid cutoff significantly influences the classification of HUA and its association with metabolic and cardiovascular outcomes [45]. Our sensitivity analysis using the cardiovascular-specific cutoff (≥ 333 µmol/L) proposed by the URRAH study [28, 29], demonstrated that NPAR maintained robust predictive performance across different uric acid thresholds, reinforcing its utility as a prognostic marker across the spectrum of uric acid elevation. This is particularly relevant given recent evidence that metabolic syndrome associations with UA vary substantially depending on the cutoff employed [45]. The stratified analysis further revealed that NPAR–mortality association was present in both clinical subgroups, though the effect for CVD mortality was attenuated in gout patients after full covariate adjustment, likely due to the limited number of CVD events (*n* = 40) in this subgroup. This finding underscores the importance of considering clinical phenotype when interpreting NPAR as a prognostic marker. Furthermore, the competing risks analysis using the Fine-Gray model confirmed that the association between NPAR and CVD mortality remained robust even after accounting for non-CVD death as a competing event, strengthening the evidence for NPAR as an independent predictor of cardiovascular outcomes in patients with HUA and gout.

## Strengths and limitations

This study establishes NPAR as an independent mortality predictor in patients with HUA and gout, especially in men and those with hypertension, attributed to the imbalance between inflammation, nutrition, and endothelial injury. NPAR can be integrated into clinical risk management strategies, and it is imperative to investigate the combined effects of anti-inflammatory and uric acid-lowering therapies. Future research should prioritize validating the role of NPAR in guiding personalized treatment approaches. To our knowledge, this is the first study to examine the impact of NPAR on mortality prognosis among U.S. adults with HUA and gout, showcasing its clinical importance. One significant strength of this research is utilizing NHANES data, which is representative of the nation, along with standardized laboratory evaluations. However, limitations include lack of detailed information on specific urate-lowering agents (e.g., allopurinol, febuxostat) and anti-inflammatory therapies, the absence of an assessment of dynamic changes in NPAR, and the need for cautious application of the findings to the broader American population. These challenges are inherent in retrospective studies; therefore, future research should incorporate prospective cohorts to enhance validity.

## Conclusion

This study demonstrates that American adults with HUA and gout with elevated NPAR levels have a higher likelihood of mortality and cardiovascular issues. This association is particularly pronounced in males and is primarily mediated through inflammatory pathways. These results highlight the potential clinical utility of NPAR as a readily available biomarker for risk stratification in patients with HUA and gout, particularly among men and those with hypertension. Future research should be carefully designed as longitudinal prospective studies to validate our findings.

## Supplementary Information

Supplementary Material 1.

## Data Availability

The datasets analyzed in this study are publicly available from the National Health and Nutrition Examination Survey (NHANES) database (https://www.cdc.gov/nchs/nhanes/index.htm). The mortality data were obtained from the National Death Index ( https://www.cdc.gov/nchs/data-linkage/mortality-public ).
