# Supplementary material for: Prognostic value of the neutrophil percentage-to-albumin ratio for mortality in patients with hyperuricemia and gout
Source: BMC Cardiovasc Disord. 2026 Jul 17;26:905. doi: 10.1186/s12872-026-06251-4 (PMC13656724; doi:10.1186/s12872-026-06251-4)
Supplement: Supplementary file 1 [file 12872_2026_6251_MOESM1_ESM.docx]

**Supporting information**

**Neutrophil percentage to albumin ratio predicts all-cause mortality and cardiovascular in** **hyperuricemia and gout patients**

**Table S1.** Collinearity Diagnostics for Covariates in the Multivariable Cox Regression Model

| **Variable** | **GVIF** | **Df** | **GVIF^(1/(2×Df))** |
| --- | --- | --- | --- |
| Age (years) | 1.291 | 1 | 1.136 |
| Gender | 1.502 | 1 | 1.052 |
| Race/Ethnicity | 1.236 | 4 | 1.112 |
| Body mass index (kg/m²) | 1.321 | 2 | 1.072 |
| Education level | 1.301 | 2 | 1.068 |
| Marital status | 1.282 | 2 | 1.064 |
| Poverty-income ratio (PIR) | 1.225 | 2 | 1.052 |
| Smoking | 1.101 | 1 | 1.049 |
| Alcohol consumption | 2.727 | 1 | 1.651 |
| Type 2 diabetes mellitus | 2.447 | 1 | 1.564 |
| Hypertension | 1.423 | 1 | 1.193 |
| Cardiovascular disease | 1.079 | 1 | 1.039 |
| Cancer history | 1.192 | 1 | 1.092 |
| Health insurance | 2.589 | 1 | 1.609 |
| Antidiabetic medication | 2.603 | 1 | 1.613 |
| Mean systolic blood pressure (mmHg) | 1.316 | 1 | 1.147 |
| Mean diastolic blood pressure (mmHg) | 8.053 | 1 | 2.838 |
| Total cholesterol (mg/dL) | 7.941 | 1 | 2.818 |
| LDL cholesterol (mg/dL) | 2.644 | 1 | 1.626 |
| Fasting glucose (mmol/L) | 3.228 | 1 | 1.797 |
| Glycohemoglobin (HbA1c, %) | 1.748 | 1 | 1.322 |
| eGFR (mL/min/1.73m²) | 1.49 | 1 | 1.221 |
| ACE inhibitor/ARB medication | 1.442 | 1 | 1.201 |
| Beta-blocker medication | 1.183 | 1 | 1.087 |
| Calcium channel blocker medication | 1.519 | 1 | 1.232 |
| Diuretic medication | 1.585 | 1 | 1.259 |
| Statin medication | 1.183 | 1 | 1.088 |
| Antiplatelet medication | 1.102 | 1 | 1.05 |
| Urate-lowering therapy (ULT) | 1.048 | 1 | 1.024 |
| Rheumatic-disease | 1.132 | 1 | 1.064 |
| Survey year | 1.291 | 1 | 1.136 |

**Abbreviations:** GVIF, generalized variance inflation factor; Df, degrees of freedom; HBP, hypertension; T2DM, type 2 diabetes mellitus; HbA1c, glycated hemoglobin; SBP, systolic blood pressure; DBP, diastolic blood pressure; eGFR, estimated glomerular filtration rate; ACEi, angiotensin-converting enzyme inhibitor; ARB, angiotensin receptor blocker; CCB, calcium channel blocker; PIR, poverty income ratio; ULT, urate-lowering therapy.

**Table S2.** Sensitive analysis to evaluate the association between NPAR with the mortality outcomes of adults with hyperuricemia and gout by excluding participants who died within 2 years.

|  |  | **All-cause mortality** |  | **CVD mortality** |  |
| --- | --- | --- | --- | --- | --- |
| **Model** | **Quartile** | **HR (95% CI)** | **P** | **HR (95% CI)** | **P** |
| **Model 1** | Q1 | 1.00 (Ref.) | — | 1.00 (Ref.) | — |
|  | Q2 | **1.62 (1.18–2.22)** | **0.003** | 1.22 (0.70–2.13) | 0.476 |
|  | Q3 | **1.84 (1.33–2.53)** | **<0.001** | 1.65 (0.96–2.83) | 0.071 |
|  | Q4 | **3.82 (2.77–5.27)** | **<0.001** | **5.06 (3.28–7.81)** | **<0.001** |
| **Model 2** | Q1 | 1.00 (Ref.) | — | 1.00 (Ref.) | — |
|  | Q2 | **1.42 (1.10–1.84)** | **0.008** | 1.07 (0.62–1.84) | 0.816 |
|  | Q3 | 1.23 (0.93–1.63) | 0.149 | 1.08 (0.65–1.79) | 0.772 |
|  | Q4 | **2.30 (1.70–3.12)** | **<0.001** | **3.11 (2.01–4.82)** | **<0.001** |
| **Model 3** | Q1 | 1.00 (Ref.) | — | 1.00 (Ref.) | — |
|  | Q2 | **1.39 (1.07–1.79)** | **0.012** | 1.07 (0.62–1.85) | 0.8 |
|  | Q3 | 1.13 (0.85–1.50) | 0.415 | 0.99 (0.59–1.67) | 0.963 |
|  | Q4 | **2.02 (1.48–2.75)** | **<0.001** | **2.75 (1.78–4.27)** | **<0.001** |
| **Model 4** | Q1 | 1.00 (Ref.) | — | 1.00 (Ref.) | — |
|  | Q2 | 1.18 (0.83–1.68) | 0.352 | 0.57 (0.29–1.10) | 0.093 |
|  | Q3 | 0.85 (0.61–1.19) | 0.348 | 0.86 (0.42–1.79) | 0.69 |
|  | Q4 | **1.72 (1.22–2.43)** | **0.002** | **2.18 (1.25–3.79)** | **0.006** |

Abbreviations: HR, hazard ratio; CI, confidence interval; NPAR, neutrophil percentage-to-albumin ratio; CVD, cardiovascular disease.

Model 1: Adjusted for NPAR and survey cycle. Model 2: Additionally adjusted for age, sex, race, BMI, education, marital status, and PIR. Model 3: Additionally adjusted for smoking, alcohol, diabetes, hypertension, CVD, cancer, insurance, antihypertensive, and antidiabetic agents. Model 4: Additionally adjusted for systolic BP, diastolic BP, total cholesterol, LDL-C, fasting glucose, HbA1c, eGFR, ACEI/ARBs, β-blockers, CCBs, diuretics, statins, antiplatelet agents, ULT, and rheumatic disease.

Note: Bold values indicate p < 0.05. Q1 served as the reference group.

**Table S3.** Sensitive analysis to evaluate the association between NPAR with the mortality outcomes of adults with hyperuricemia and gout by only including participants from 1999 to 2006.

|  |  | **All-cause mortality** |  | **CVD mortality** |  |  |
| --- | --- | --- | --- | --- | --- | --- |
| **Model** | **Quartile** | **HR (95% CI)** | ***P*** | **HR (95% CI)** | ***P*** |  |
| **Model 1** | Q1 | 1.00 (Ref.) | — | 1.00 (Ref.) | — |  |
|  | Q2 | **1.62 (1.04–2.54)** | **0.034** | 1.34 (0.66–2.74) | 0.423 |  |
|  | Q3 | 1.56 (0.96–2.56) | 0.075 | 1.23 (0.59–2.56) | 0.581 |  |
|  | Q4 | **3.60 (2.27–5.72)** | **<0.001** | **4.31 (2.29–8.10)** | **<0.001** |  |
| **Model 2** | Q1 | 1.00 (Ref.) | — | 1.00 (Ref.) | — |  |
|  | Q2 | **1.53 (1.04–2.25)** | **0.032** | 1.11 (0.54–2.29) | 0.77 |  |
|  | Q3 | 1.11 (0.74–1.67) | 0.612 | 0.85 (0.44–1.65) | 0.629 |  |
|  | Q4 | **2.20 (1.38–3.49)** | **<0.001** | **2.66 (1.36–5.20)** | **0.004** |  |
| **Model 3** | Q1 | 1.00 (Ref.) | — | 1.00 (Ref.) | — |  |
|  | Q2 | 1.44 (0.96–2.17) | 0.082 | 1.06 (0.48–2.35) | 0.89 |  |
|  | Q3 | 1.06 (0.71–1.60) | 0.777 | 0.77 (0.37–1.59) | 0.477 |  |
|  | Q4 | **1.90 (1.15–3.14)** | **0.012** | **2.20 (1.03–4.69)** | **0.042** |  |
| **Model 4** | Q1—Q4 | — | — | — | — |  |
| Model 4 could not be estimated due to insufficient events for the number of covariates (N = 1,176). | | | | | |  |
|  |  |  |  |  |  |  |

Abbreviations: HR, hazard ratio; CI, confidence interval; NPAR, neutrophil percentage-to-albumin ratio; CVD, cardiovascular disease.

Model 1: Adjusted for NPAR and survey cycle. Model 2: Additionally adjusted for age, sex, race, BMI, education, marital status, and PIR. Model 3: Additionally adjusted for smoking, alcohol, diabetes, hypertension, CVD, cancer, insurance, antihypertensive, and antidiabetic agents. Model 4: Additionally adjusted for systolic BP, diastolic BP, total cholesterol, LDL-C, fasting glucose, HbA1c, eGFR, ACEI/ARBs, β-blockers, CCBs, diuretics, statins, antiplatelet agents, ULT, and rheumatic disease.

Note: Bold values indicate p < 0.05. Q1 served as the reference group.

**Table S4.** HRs (95% CIs) and *P* values for all-cause and cardiovascular mortality by NPAR quartiles, stratified by serum uric acid cutoffs.

| **UA cutoff** | **Outcome** | **Model** | **Q2 vs Q1** | **Q3 vs Q1** | **Q4 vs Q1** | ***P* for trend** |
| --- | --- | --- | --- | --- | --- | --- |
| **≥286 μmol/L** | All-cause mortality | M1 | 1.43 (1.17–1.75) | 1.98 (1.66–2.36) | 4.01 (3.32–4.85) | <0.001 |
|  |  | M2 | 1.12 (0.92–1.37) | 1.20 (0.97–1.49) | 1.92 (1.60–2.30) | <0.001 |
|  |  | M3 | 1.02 (0.80–1.31) | 1.04 (0.80–1.35) | 1.85 (1.45–2.37) | <0.001 |
|  |  | M4 | 1.16 (0.80–1.67) | 0.94 (0.64–1.38) | 1.55 (1.11–2.16) | 0.011 |
|  | CVD mortality | M1 | 1.19 (0.71–1.99) | 2.11 (1.33–3.35) | 5.48 (3.60–8.35) | <0.001 |
|  |  | M2 | 1.08 (0.64–1.83) | 1.31 (0.87–1.98) | 2.68 (1.77–4.03) | <0.001 |
|  |  | M3 | 0.64 (0.30–1.37) | 1.17 (0.64–2.16) | 2.33 (1.43–3.81) | <0.001 |
|  |  | M4 | 0.75 (0.34–1.64) | 1.15 (0.56–2.37) | 2.09 (1.18–3.70) | 0.011 |
| **≥333 μmol/L** | All-cause mortality | M1 | 1.63 (1.29–2.06) | 2.16 (1.67–2.79) | 4.71 (3.72–5.95) | <0.001 |
|  |  | M2 | 1.26 (0.90–1.75) | 1.23 (0.94–1.62) | 2.08 (1.57–2.76) | <0.001 |
|  |  | M3 | 1.29 (0.86–1.95) | 1.14 (0.75–1.74) | 1.99 (1.40–2.84) | <0.001 |
|  |  | M4 | 1.13 (0.77–1.65) | 0.91 (0.62–1.35) | 1.53 (1.09–2.16) | 0.014 |
|  | CVD mortality | M1 | 1.43 (0.81–2.50) | 2.37 (1.42–3.96) | 6.62 (4.31–10.17) | <0.001 |
|  |  | M2 | 1.28 (0.76–2.17) | 1.38 (0.85–2.25) | 2.89 (1.89–4.43) | <0.001 |
|  |  | M3 | 0.80 (0.37–1.71) | 1.21 (0.66–2.23) | 2.27 (1.22–4.23) | 0.010 |
|  |  | M4 | 0.75 (0.34–1.66) | 1.13 (0.55–2.32) | 2.08 (1.17–3.70) | 0.012 |
| **Traditional*** | All-cause mortality | M1 | 1.59 (1.11–2.28) | 1.82 (1.32–2.52) | 4.19 (3.03–5.80) | <0.001 |
|  |  | M2 | 1.33 (0.94–1.89) | 1.26 (0.86–1.83) | 2.37 (1.70–3.30) | <0.001 |
|  |  | M3 | 1.34 (0.86–2.07) | 1.06 (0.69–1.65) | 1.69 (1.19–2.41) | 0.004 |
|  |  | M4 | 1.20 (0.80–1.82) | 0.88 (0.58–1.32) | 1.43 (1.00–2.04) | 0.047 |
|  | CVD mortality | M1 | 1.14 (0.52–2.50) | 1.69 (0.92–3.11) | 5.59 (3.41–9.14) | <0.001 |
|  |  | M2 | 1.13 (0.61–2.07) | 1.27 (0.59–2.72) | 3.32 (1.90–5.78) | <0.001 |
|  |  | M3 | 0.76 (0.24–2.45) | 1.31 (0.48–3.61) | 2.24 (1.10–4.56) | 0.026 |
|  |  | M4 | 0.86 (0.37–2.04) | 1.28 (0.61–2.70) | 2.17 (1.19–3.95) | 0.012 |

Abbreviations: HR, hazard ratio; CI, confidence interval; NPAR, neutrophil percentage-to-albumin ratio; UA, uric acid; CVD, cardiovascular disease.

Model 1: Adjusted for NPAR and survey cycle. Model 2: Additionally adjusted for age, sex, race, BMI, education, marital status, and PIR. Model 3: Additionally adjusted for smoking, alcohol, diabetes, hypertension, CVD, cancer, insurance, antihypertensive, and antidiabetic agents. Model 4: Additionally adjusted for systolic BP, diastolic BP, total cholesterol, LDL-C, fasting glucose, HbA1c, eGFR, ACEI/ARBs, β-blockers, CCBs, diuretics, statins, antiplatelet agents, ULT, and rheumatic disease.

Note: Bold values indicate p < 0.05. Q1 served as the reference group. P for trend was calculated by entering NPAR quartiles as a continuous variable in the Cox model. *Traditional: Traditional hyperuricemia cutoff (≥420 μmol/L for men, ≥360 μmol/L for women).

**Table S5.** Incremental predictive value of NPAR across subgroups

| **Population** | **Full cohort** | | **HUA** | | **Gout** | |
| --- | --- | --- | --- | --- | --- | --- |
| **Outcome** | All‑cause | CVD | All‑cause | CVD | All‑cause | CVD |
| **N (events)** | 4,344 (887) | 4,344 (268) | 3,659 (736) | 3,659 (228) | 685 (151) | 685 (40) |
| **ΔC-index** | 0.010 | 0.016 | 0.008 | 0.012 | 0.029 | 0.025 |
| **IDI** | 0.034 (<0.001) | 0.032 (<0.001) | 0.028 (<0.001) | 0.028 (<0.001) | 0.066 (<0.001) | 0.083 (0.04) |
| **NRI** | 0.197 (<0.001) | 0.311 (<0.001) | 0.205 (<0.001) | 0.309 (<0.001) | 0.227 (<0.001) | 0.377 (0.04) |
| **Median-Improvement** | 0.015 (<0.001) | 0.008 (<0.001) | 0.013 (<0.001) | 0.008 (<0.001) | 0.025 (0.06) | 0.039 (0.159) |

**Abbreviations:** NPAR, number of participants at risk; HUA, hyperuricemia; CVD, cardiovascular disease; NRI, net reclassification improvement; IDI, integrated discrimination improvement.

**Note:** The base model included age, sex, race/ethnicity, body mass index, education level, marital status, poverty‑income ratio, smoking status, alcohol consumption, diabetes, hypertension, cardiovascular disease, cancer, and health insurance. The full model comprised the base model plus NPAR as a continuous variable. All metrics were derived from 5‑year risk predictions. ΔC‑index = difference in concordance index between the full and base models

**Figure S1.** The distribution of the continuous variables in this study. BMI: body mass index; NPAR：neutrophil-percentage-to-albumin ratio.

**Figure S2.** Association between NPAR with all-cause mortality among adults with hyperuricemia and gout patients in different subgroups. NPAR: neutrophil percentage-to-albumin ratio; BMI: body mass index; PIR: poverty income ratio; CVD: cardiovascular disease; DM: diabetes mellitus; HBP: hypertension.

**Figure S3.** Association between NPAR with Cardiovascular mortality among adults with hyperuricemia and gout patients in different subgroups. NPAR：neutrophil-percentage-to-albumin ratio; BMI: body mass index; PIR: poverty income ratio; DM: diabetes mellitus; HBP: hypertension.

**Figure S4.** Dose-response association between NPAR and all-cause mortality, stratified by asymptomatic hyperuricemia and gout.

Restricted cubic spline curves with 3 knots (at the 10th, 50th, and 90th percentiles) were used to model the relationship between NPAR and all-cause mortality among NHANES participants, stratified by clinical subgroup. The left panel shows participants with asymptomatic hyperuricemia (n = 3,659); the right panel shows participants with gout (n = 685). Solid lines represent hazard ratios, and shaded areas indicate 95% confidence intervals, with the reference point (HR = 1) set at the median NPAR level within each subgroup. Four sequential models are presented: Model 1 (crude, red), Model 2 (adjusted for age, sex, race/ethnicity, BMI, education, marital status, and poverty‑income ratio, blue), Model 3 (additionally adjusted for smoking, alcohol, diabetes, hypertension, CVD, cancer, insurance, antihypertensive, and antidiabetic medications, orange), and Model 4 (fully adjusted, green).

**Figure S5.** Dose-response association between NPAR and cardiovascular mortality, stratified by asymptomatic hyperuricemia and gout.

Restricted cubic spline curves with 3 knots (at the 10th, 50th, and 90th percentiles) were used to model the relationship between NPAR and cardiovascular mortality among NHANES participants, stratified by clinical subgroup. The left panel shows participants with asymptomatic hyperuricemia (n = 3,659); the right panel shows participants with gout (n = 685). Solid lines represent hazard ratios, and shaded areas indicate 95% confidence intervals, with the reference point (HR = 1) set at the median NPAR level within each subgroup. Four sequential models are presented: Model 1 (crude, red), Model 2 (adjusted for age, sex, race/ethnicity, BMI, education, marital status, and poverty‑income ratio, blue), Model 3 (additionally adjusted for smoking, alcohol, diabetes, hypertension, CVD, cancer, insurance, antihypertensive, and antidiabetic medications, orange), and Model 4 (fully adjusted, green).

**Figure S6.** Kaplan–Meier survival curves for all‑cause and cardiovascular mortality by NPAR quartiles in the asymptomatic hyperuricemia subgroup

Panel A shows all‑cause mortality‑free survival; Panel B shows cardiovascular mortality‑free survival. Participants were stratified by NPAR quartiles (Q1–Q4). The log‑rank test was used to compare survival distributions across groups. Shaded areas represent 95% confidence intervals. The numbers at risk at each time point are presented below the curves. Kaplan–Meier survival curves for (A) all‑cause mortality and (B) cardiovascular mortality, stratified by NPAR quartiles (Q1–Q4) among participants with asymptomatic hyperuricemia (n = 3,659). Survival distributions were compared using the log‑rank test. The numbers of participants at risk at each time point are presented below the curves.

**Figure S7.** Kaplan–Meier survival curves for all‑cause and cardiovascular mortality by NPAR quartiles in the gout subgroup.

Kaplan–Meier survival curves for (A) all‑cause mortality and (B) cardiovascular mortality, stratified by NPAR quartiles (Q1–Q4) among participants with gout (n = 685). Survival distributions were compared using the log‑rank test. The numbers of participants at risk at each time point are presented below the curves.

**Figure S8**. Directed acyclic graph (DAG) illustrating the hypothesized causal relationships between NPAR and mortality.

The exposure variable (NPAR) is shown in red, and the outcome (mortality) in dark grey. Blue nodes represent core confounders adjusted for in Model 3: age (RIDAGEYR), sex (RIAGENDR), race/ethnicity (Race_N), BMI (BMXBMI), education level, marital status, poverty-income ratio (PIR_gr), survey year, smoking status, alcohol consumption, type 2 diabetes mellitus (TDM), hypertension (HBP), cardiovascular disease (CVD), cancer history, and health insurance status. Orange nodes represent potential mediators added in Model 4: mean systolic blood pressure (meanSBP), mean diastolic blood pressure (meanDBP), total cholesterol (LBXTC), LDL cholesterol (LBDLDL), fasting glucose (LBDGLUSI), glycohemoglobin (LBXGH), and estimated glomerular filtration rate (eGFR). Purple nodes represent medication use and competing exposures added in Model 4: ACE inhibitor/ARB (ACEI_ARB), β-blocker, calcium channel blocker (CCB), diuretics, statin, antiplatelet agents, antidiabetic medication, urate-lowering therapy (ULT), and rheumatic disease. Arrows indicate hypothesized causal directions. Model 3 adjusts for all blue variables. Model 4 additionally adjusts for orange, purple, and grey variables (indirect pathways). The DAG was constructed based on clinical knowledge and prior literature to support covariate selection for the multivariable Cox models and to distinguish between confounders, mediators, and competing exposures.

**Figure S9**. Cumulative incidence of CVD and non‑CVD death by NPAR quartiles — Fine‑Gray competing risk model.

Cumulative incidence functions for CVD death (left panel) and non‑CVD death (right panel) were estimated using the Fine‑Gray subdistribution hazard model, with non‑CVD death specified as the competing event. Participants were stratified by NPAR quartiles: Q1 (NPAR ≤ 12.20), Q2 (12.20 < NPAR ≤ 13.89), Q3 (13.90 < NPAR ≤ 15.69), and Q4 (NPAR ≥ 15.70). Shaded areas represent 95% confidence intervals. Cumulative incidence values at 5 and 10 years are annotated on the curves. The numbers at risk at each time point are presented in the table below. Survival distributions were compared using Gray’s test. Median follow-up: 95 months in the competing risk analysis subset.

**Figure S10**. Fine‑Gray competing risk model for CVD mortality by NPAR quartiles

Fine‑Gray competing risk model for CVD mortality by NPAR quartiles. Subdistribution hazard ratios (sHR) were estimated using the Fine‑Gray model via the cause‑specific Cox approach with data duplication, stratified by event type, and weighted by NHANES survey design. Four progressive models were constructed: Model 1, crude; Model 2, adjusted for demographics (age, sex, race, BMI, education, marital status, PIR); Model 3, additionally adjusted for lifestyle factors and comorbidities (smoking, alcohol, diabetes, hypertension, CVD, cancer, insurance); Model 4, fully adjusted additionally including laboratory measurements (SBP, DBP, total cholesterol, LDL, glucose, HbA1c, eGFR) and medications (ACEi/ARB, β-blocker, CCB, diuretics, statin, antiplatelet, antidiabetic, ULT) and rheumatic disease. Error bars represent 95% confidence intervals. The dashed vertical line indicates the null value (sHR = 1.0).

**Figure S11**. Fine-Gray subdistribution hazard ratios (SHRs) with 95% confidence intervals (CIs) for cardiovascular disease (CVD) mortality in the competing risks model

The neutrophil percentage-to-albumin ratio (NPAR) was analyzed as quartiles, with Q1 as the reference. Significant associations (P < 0.05) are indicated by bold text and square markers. SHR, subdistribution hazard ratio; CI, confidence interval; NPAR, neutrophil percentage-to-albumin ratio; BP, blood pressure; eGFR, estimated glomerular filtration rate; ACEi, angiotensin-converting enzyme inhibitor; ARB, angiotensin receptor blocker; CCB, calcium channel blocker.
